# The effect of LHC jet data on MSTW PDFs

**DOI:** 10.1140/epjc/s10052-014-2934-z

**Published:** 2014-07-01

**Authors:** B. J. A. Watt, P. Motylinski, R. S. Thorne

**Affiliations:** Department of Physics and Astronomy, University College London, Gower Place, London, WC1E 6BT UK

## Abstract

We consider the effect on LHC jet cross sections on partons distribution functions (PDFs), in particular the MSTW2008 set of PDFs. We first compare the published inclusive jet data to the predictions using MSTW2008, finding a very good description. We also use the parton distribution reweighting procedure to estimate the impact of these new data on the PDFs, finding that the combined ATLAS 2.76 and 7 TeV data, and CMS 7 TeV data have some significant impact. We then also investigate the impact of ATLAS, CMS and DØ dijet data using the same techniques. In this case we investigate the effect of using different scale choices for the NLO cross section calculation. We find that the dijet data is generally not completely compatible with the corresponding inclusive jet data, often tending to pull PDFs, particularly the gluon distribution, away from the default values. However, the effect depends on the dijet dataset used as well as the scale choice. We also note that conclusions may be affected by limiting the pull on the data luminosity chosen by the best fit, which is sometimes a number of standard deviations. Finally we include the inclusive jet data in a new PDF fit explicitly. This enables us to check the consistency of the exact result with that obtained from the reweighting procedure. There is generally good, but not full quantitative agreement. Hence, the conclusion remains that MSTW2008 PDFs already fit the published jet data well, but the central values and uncertainties are altered and improved, respectively, to a significant, but not dramatic extent by inclusion of these data.

## Introduction

When considering hadron collider data for the determination of PDFs, one of the most effective and distinguishing sets is the cross section for production of high-$p_T$ jets. Indeed, this is one of the few direct probes of the gluon distribution in PDF fits, with the gluon constraint from fitting DIS and Drell–Yan data being overwhelmingly indirect via the quark and antiquark evolution. Until recently, the only hadron collider data on the inclusive jet cross section which was available for PDF fits was that produced at the Tevatron by the CDF [1] and DØ [2] Collaborations. These were shown to have a significant constraining effect on the PDFs, and were particularly useful in decoupling the correlation between the gluon distribution and the strong coupling. The introduction of LHC data is expected to have an even larger impact on the current modern PDF sets [3–7] due to the extension in the range of $x$ and $Q^2$ probed. We will consider the quality of the fit to LHC inclusive jet data, both from ATLAS and CMS, in Sect. 2 of this article. As well as investigating how well the current MSTW PDFs fit the data we will examine the impact of the data both by considering the PDF uncertainty eigenvectors and checking which improve the fit quality and which cause it to deteriorate, and also by using the PDF reweighting procedure. The latter provides a quantitative estimate of the genuine effect of a new dataset on both the central value and the uncertainty of a PDF set.

So far the only type of hadron collider jet data included in the determination of the MSTW 2008 PDF sets, or indeed any other available PDF set, is the inclusive jet production. There is some Tevatron dijet data [8] spanning the same range in energy and rapidity as the inclusive data, but this has not been used in used obtaining PDFs, though some studies of the fit quality and potential impact have been made [9, 10]. This is perhaps largely due to the fact that the dijet data sample has a significant overlap with the inclusive jet data sample. The inclusive data were chosen due to there being a less reliable theoretical understanding of the high-rapidity dijet production as a function of dijet mass, $M_\mathrm{JJ}$. This issue will be studied in more detail in Sect. 3 of this article, for both the older DØ dijet data and the more recent ATLAS and CMS data. As for the inclusive data the fit quality using the existing MSTW2008 PDFs, and the potential impact of the new data will be studied.

In the next section we will include the ATLAS and CMS inclusive data in a new fit explicitly using the MSTW2008 framework. This will provide the most detailed results on the impact of these new data sets, also including the effect on the strong coupling constant $\alpha _\mathrm{S}(M_Z^2)$ obtained from the fit. It also provides an opportunity to compare the results from including a new dataset explicitly in the fit with the results obtained from PDF reweighting, the first time this has been studied for the reweighting procedure using the Hessian approach. We find reasonable agreement between the results obtained using reweighting and from fitting explicitly, but the former seems to imply a slightly greater reduction in uncertainty than is found from direct inclusion of data. We also briefly investigate different forms of reweighting and the uncertainty estimation for PDFs.

Throughout this article we will base our main results on an analysis using PDFs and jet cross sections at NLO in QCD, removing any ambiguity due to the lack of knowledge of the full NNLO jet cross sections. We will comment on NNLO corrections at the end of the article. There are also electroweak corrections (see [11] for a summary) which could potentially be quite large. However, there is still some disagreement upon the nature of these corrections, and so they are omitted from the analysis. We also base our study on the framework of the MSTW2008 PDF fit. Although there have been updates [12–14] including new datasets, PDF parameterisations, deuterium corrections and heavy flavour schemes, and even some publicly released PDFs [12], in order to isolate the singular effect of the inclusion of jet data without potential contamination from these other updates we present the impact of the jet data and nothing else on MSTW2008 PDFs. A forthcoming PDF update will include all these various sources of improvement or update, and the specific impact of the jet data within this larger set of changes is known to be very similar to that presented in this article.

## Inclusive jets

**Fig. 1:**
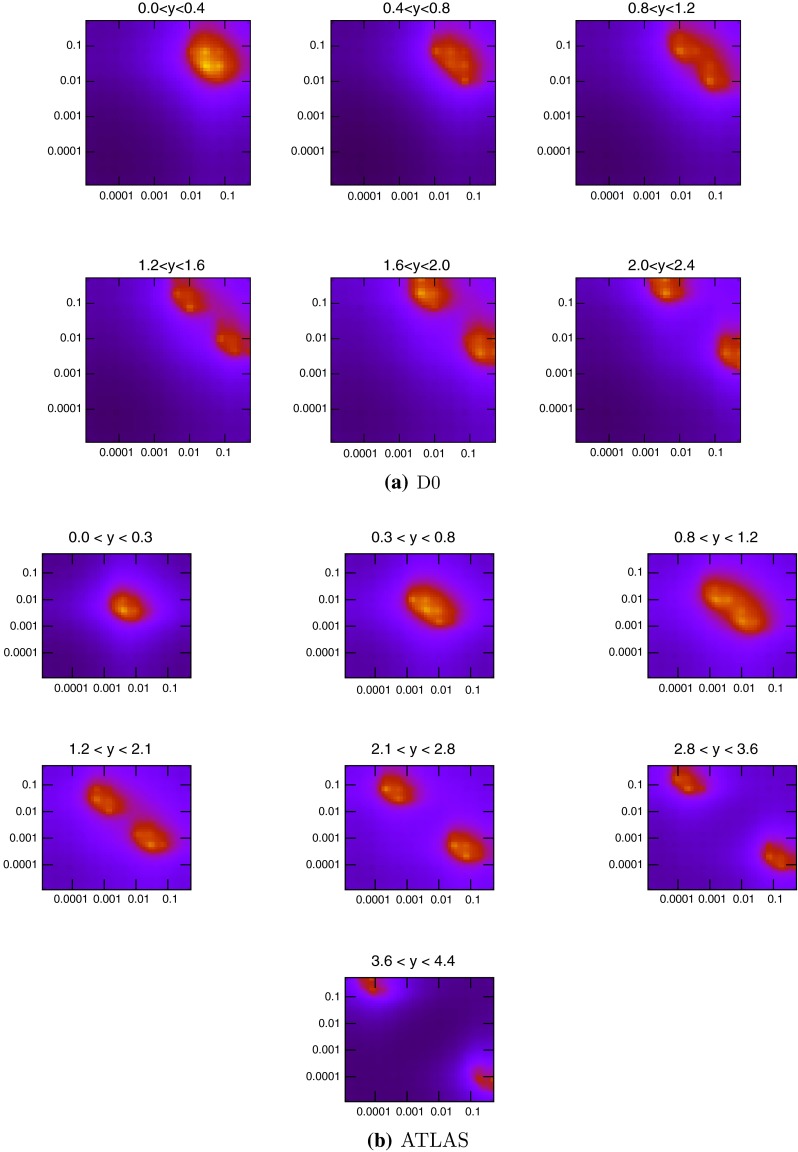
Values of $x_1$ (*higher x*) and $x_2$ (*lower x*) for each event generated in NLOJet++ for inclusive jets at the Tevatron ($\sqrt{s}=1.96$ TeV) and LHC ($\sqrt{s}=7$ TeV). The lowest-$p_T$ jets dominate in each rapidity bin, so the higher values of $x$ probed at large $p_T$ do not appear

**Fig. 2:**
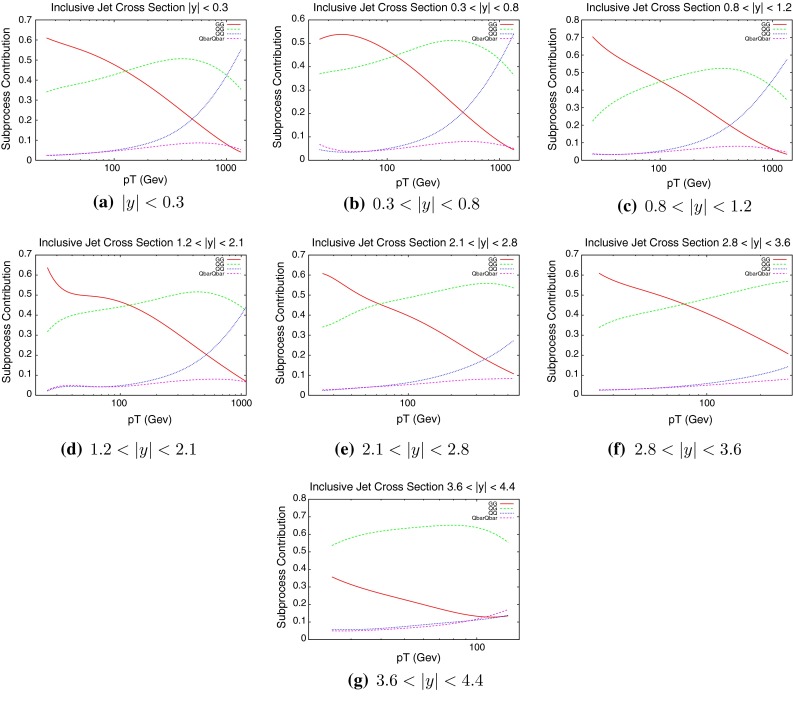
Contributions of different initial-state parton combinations to the inclusive jet cross section calculation at ATLAS

In this section the details of the theoretical prediction for inclusive jet cross sections at the LHC are studied and the effects they have on the PDFs is analysed. The first LHC data to have a true ability to probe new regions of the $(x,Q^2)$ plane for current PDFs was that from the ATLAS Collaboration on the inclusive jet and dijet cross sections at 7 TeV using 36 pb$^{-1}$ of data [15]. To demonstrate this ability, Fig. 1 shows the distribution of the parton momentum fractions $x_1$ and $x_2$ for NLOJet++ [16, 17] events at the Tevatron and the LHC. In these, and similar subsequent plots, the points have been generated at NLO using unweighted events, though the plots would look very similar at LO. In the highest-rapidity bin, the ATLAS data is probing values of $x\approx 10^{-5}$, two orders of magnitude lower than at DØ. These plots are dominated by the low-$p_T$ bins within each rapidity bin, due to the orders of magnitude greater number of jets produced at low $p_T$. The higher-$p_T$ jets require higher $x$ values, and the spots in Fig. 1 shift along the diagonal line parallel to $x_1=x_2$ towards higher $x$ as the $p_T$ of the jets is increased. Comparing the plots at LHC and Tevatron energies shows the value of the LHC data. For inclusive jets, the PDFs can be probed down to $x=10^{-5}$ at high rapidities, a factor of 10 better than the Tevatron reach. The sensitivity of the data to different partons is demonstrated in Fig. 2, where the cross section calculation is broken down into four partonic subprocesses: gluon–gluon, quark–gluon, quark–quark, antiquark–antiquark.

Clearly, different areas of phase space provide more information about certain PDFs than others. In the lowest-rapidity bin for instance, the low-$p_T$ jets are produced predominantly by initial state gluons, whereas the hardest jets are dominated by the quark–quark process. By combining this information with that obtained from Fig. 1, we can see that the low-$p_T$ central jets will provide information on the low-*x* gluon, whereas high-$p_T$ will shed light on the high-*x* valence quark distributions. The fraction probed changes as a function of rapidity. As the rapidity of the inclusive jets increases, the events are produced predominantly by a combination of one low-$x$ and one high-$x$ parton, which can again be seen in the plots of Fig. 1. This means that the quark–gluon process becomes dominant at high rapidities, especially at high $p_T$, and so these bins in the data will simultaneously probe the gluon and the quark distributions.

The $\chi ^2$ used to compare data to theory is similar to that used for jet data in MSTW PDF fits. Each data point is allowed to move with respect to the theory prediction due to the many systematic uncertainties in the measurement. For each source of systematic uncertainty, a nuisance parameter $r_k$ is introduced, such that shifts will only occur if the reduction in $\chi ^2$ is significant. The exact form of the expression used is

1 $$\begin{aligned} \chi ^2=\sum _{i=1}^{N_\mathrm{pts}}\left( \frac{D_i-\sum _{k=1}^{N_\mathrm{corr}}r_k\sigma _{k,i}^\mathrm{corr}-T_i}{\sigma _i^\mathrm{uncorr}}\right) ^2+\sum _{k=1}^{N_\mathrm{corr}}r_k^2 \end{aligned}$$

where $i$ labels the individual data points and $k$ labels the correlated systematics. For the ATLAS 7 TeV data, the number of correlated systematics is 88 when including the hadronisation uncertainty. The uncorrelated error is the sum in quadrature of the statistical error and the three uncorrelated systematic errors. This definition is not identical to the standard MSTW fit due to the treatment of normalisations, which here is considered a standard source of systematic error. In the actual fits, the normalisations are treated separately, and this will be discussed later in the article.

It is possible to solve this equation for $r_k$ analytically, giving the optimum systematic shifts directly. By minimising $\chi ^2$ the result is

2 $$\begin{aligned} r_k=\sum _{k'=1}^{N_\mathrm{corr}}(A^{-1})_{kk'}B_{k'} \end{aligned}$$

where

3 $$\begin{aligned} A_{kk'}=\delta _{kk'}+\sum _{i=1}^{N_\mathrm{pts}}\frac{\sigma ^\mathrm{corr}_{k,i}\sigma ^\mathrm{corr}_{k',i}}{(\sigma ^\mathrm{uncorr}_i)^2},\quad B_k{=}\sum _{i=1}^{N_\mathrm{pts}}\frac{\sigma ^\mathrm{corr}_{k,i}(D_i-T_i)}{(\sigma ^\mathrm{uncorr}_i)^2}. \end{aligned}$$

Hence, by calculating and subsequently inverting the $88\times 88$ matrix $A$, and the vector $B$, the optimal values of the nuisance parameters can be found.

The correlated systematics for both the inclusive and the dijet data sets are mostly antisymmetric, and so a method of symmetrising to obtain a single error for each data point must be employed. Since this is a matter of choice and should not affect the results in any meaningful way, three opposing methods were used to test the effect. These were:

$$\begin{aligned} \sigma _\mathrm{corr}&= |{\sigma _\mathrm{corr}}^+| \\ \sigma _\mathrm{corr}&= |{\sigma _\mathrm{corr}}^-| \\ \sigma _\mathrm{corr }&= \frac{(|{\sigma _\mathrm{corr}}^+| + |{\sigma _\mathrm{corr}}^-|)}{2}. \end{aligned}$$

${\sigma _\mathrm{corr}}^{+/-}$ denotes the two opposing values of the antisymmetric errors, and the convention is that the sign denotes the sign of the error for the first point, i.e. lowest rapidity and $p_T$ (or $M_\mathrm{JJ}$ for dijets). The sign of the error is not necessarily maintained throughout the whole dataset. The difference in $\chi ^2$ obtained from these methods varied by no more than $3~\%$ across all theory predictions. In all the following results, the third definition is used to calculate the $\chi ^2$ values.

### The effect of the ATLAS inclusive jet data at 7 TeV

**Fig. 3:**
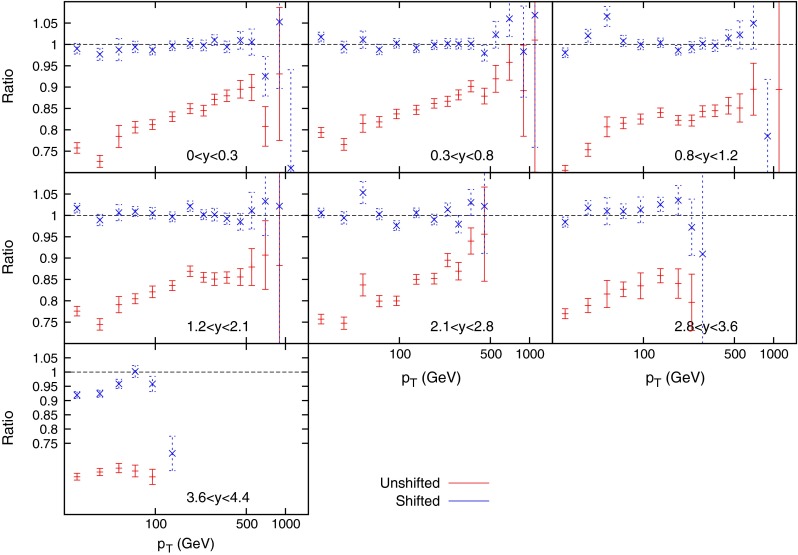
Ratio of data to theory for ATLAS inclusive jets ($R=0.4$)

Figure 3 shows the ratio of data to theory (calculated with FastNLO [18] version 2 [19] which uses NLOJet++ [16, 17]) using MSTW2008 NLO PDFs for the ATLAS 7 TeV *R* = 0.4 inclusive jet cross section, both before and after the correlated systematics are taken into account. The former gives a very poor agreement, with all data points above theory by up to 40 %. The systematics are, however, large and the shifted points, defined as $(D_i-\sum _{k=1}^{N_\mathrm{corr}}r_k\sigma _{k,i}^\mathrm{corr})/T_i$ are almost all within 1$\sigma$ of 1. The $R=0.4$ dataset is chosen as default over the $R=0.6$ due to the much smaller hadronisation corrections in the case of the smaller jet parameter. Tables 1 and 2 demonstrate that a $\chi ^2$ of less than 1 per point is achieved for a variety of scale choices and both R parameter choices, whilst the vast majority of the $r_k$’s penalty terms are less than 0.5. This implies that the fit is a very good one, however, the large shift observed in the data alongside the small penalty terms implies that the systematic uncertainties are very large, and may be drowning out any underlying physics effects.

**Table 1:** $\chi ^2$ per point (90 points)

Scale	$p_T$/2	$p_T$	2$p_T$
$R=0.4$	0.75	0.78	0.70
$R=0.6$	0.85	0.79	0.72

**Table 2:** Distribution of the $r_k$ (total 88)

$$|r_k| $$	$${<}0.5$$	0.5–1.5	1.5–2.5	2.5–3.5
$R=0.4$	72	15	1	0
$R=0.6$	74	13	1	0

**Fig. 4:**
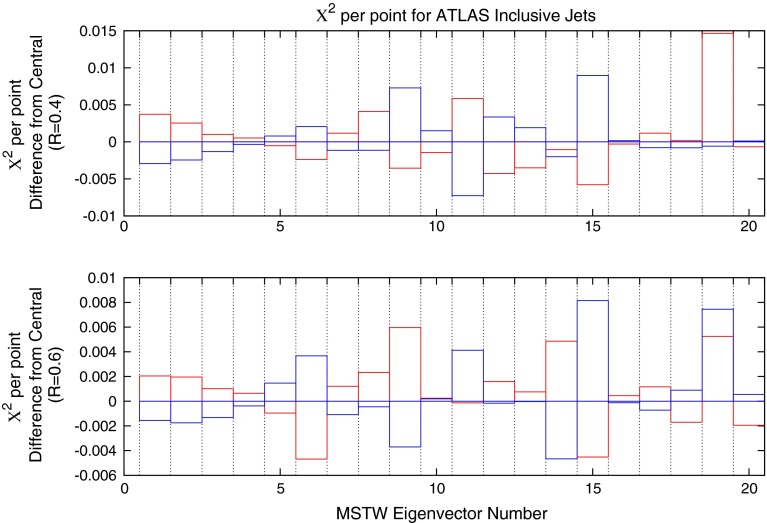
Change in fit quality for each MSTW eigenvector direction for ATLAS inclusive jets for both *R*-parameters used. The *blue* (*red*) *bars* indicate positive (negative) movement in the eigenvector direction

A useful tool for extracting information on how the partons are affected by a new dataset is to analyse the change in fit quality when using the different eigenvector sets in a global PDF fit. The global minimum of the PDF set will not necessarily give the best fit to any individual dataset, due to competing influences from other sets used in the global fit. The overcompensation of systematic effects is further demonstrated in Fig. 4. Firstly, the individual eigenvectors are varied, and predictions produced corresponding to 1$\sigma$ deviations in each direction. The change in $\chi ^2$ is negligible for all eigenvectors, with a maximum improvement of 0.007 per point in the $R=0.4$ fit for eigenvector 11.

In order to best determine if there is any impact on the PDFs from a new dataset, another method which can be employed is the reweighting procedure. This was first suggested in [20], reintroduced and modified in [21, 22] in the context of PDFs obtained from fits to replicas of data, and then extended for use with replicas obtained from PDF eigenvectors in the Hessian approach in [23] (see also [24]). We briefly summarise the latter approach. Firstly, the prediction for each eigenvector in the MSTW2008 fit is produced. These predictions are used to produce 1,000 PDFs randomly distributed in eigenvector space, using the formula:

4 $$\begin{aligned} F(S_k) = F(S_0)+\sum _{j=1}^n [F(S_j^{\pm })-F(S_0)] |R_{jk}| \end{aligned}$$

where $R_{jk}$ is a Gaussian-distributed random number. The $F(S_k)$ can be any observable calculated using a PDF eigenvector $S_k$, in this case the jet cross sections. By sampling the eigenvector sets directly and weighting each PDF equally, an accurate estimate of the Hessian error on each data point is obtained. The central prediction is estimated simply by taking the average of these unweighted predictions. Although this does not exactly reproduce the prediction from the PDF set at the global minimum of the fit, the deviations are small and always well within the $1\!-\!\sigma$ error band. The main source of these deviations is the nonlinear dependence of the parameters on $x$. For example, if a function takes the form $(1-x)^{\eta }$ the average of $(1-x)^{\eta +\delta }$ and $(1-x)^{\eta -\delta }$ is not exactly equal to $(1-x)^{\eta }$, even if $\delta$ is small.

**Fig. 5:**
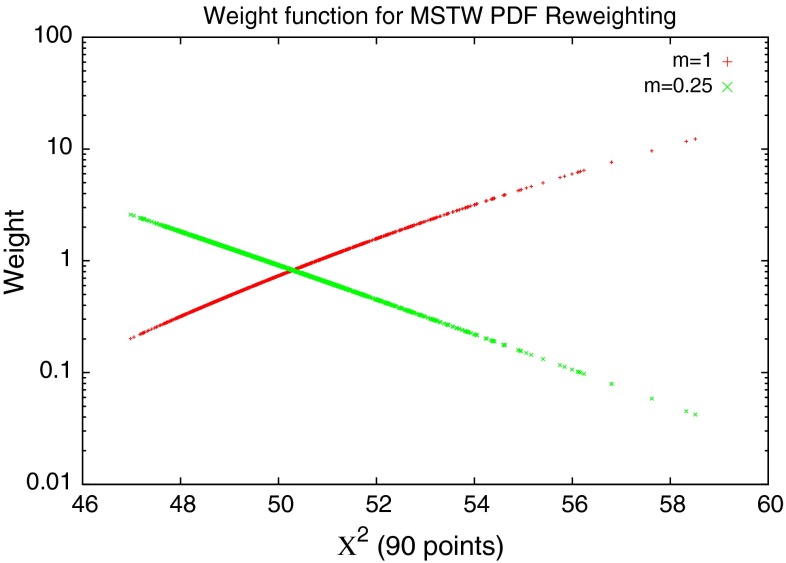
Weights for 1,000 random PDFs, each fit to a dataset of 90 points with many PDFs giving $\chi ^2$ better than $1$ per point. In this instance the standard reweighting function breaks down, and a value of $m<1$ is needed to properly weight the PDFs

In order to obtain the effect of a new dataset on the PDFs each random PDF is weighted according to its $\chi ^2$, and then the statistical combination provides an updated ideal PDF for the dataset in question. The weighting formula advocated in [21, 22] is

5 $$\begin{aligned} w_i(\chi ^2_i)&= \frac{W_i(\chi ^2_i)}{\frac{1}{N_\mathrm{pdf}}\sum _{j=1}^{N_\mathrm{pdf}} W_j(\chi ^2_j)} , \nonumber \\ W_i(\chi ^2_i)&= [\chi ^2_i]^{\frac{m*(N_\mathrm{pts}-1)}{2}}\exp \left( -\frac{\chi ^2_i}{2}\right) \end{aligned}$$

where $\chi ^2_i$ is the fit quality of the $i$th random PDF, $N_\mathrm{pdf}$ is the number of random PDFs generated and $N_\mathrm{pts}$ is the number of points in the fit, and by default $m=1$. There is some active discussion as regards the correct weighting function to use (see e.g. [25, 26]), although the most appropriate choice is certainly related to the procedure used in the PDF fit to obtain the uncertainty. Since in the MSTW fitting procedure the so-called “dynamical tolerance” procedure, based on the confidence level of the fit quality to individual datasets is used, the weighting in (5) might seem appropriate. The weighting function used here is also modified to include the multiplying factor $m$, to account for the case where the fit gives a $\chi ^2$ significantly better than $1$ per point. In this instance, the weight function has a turning point, and so assigns lower weights to the best fits than those slightly worse. This is demonstrated in Fig. 5, where all random PDFs give a better fit than $1$ per point (e.g. ATLAS inclusive jet data). In this case, a value of $m<1$ is required to ensure the weights are assigned correctly. The actual value of $m$ to choose will affect how quickly the weights decrease as the fit worsens. For the ATLAS inclusive jet data values of $m \approx 0.5$ were used since the $\chi ^2$ per point cannot be much more than 0.5. The shape of the function $[\chi ^2_i]^{\frac{m*(N_{pts}-1)}{2}}\exp (-\frac{\chi ^2_i}{2})$ does not change much for variations of $m$ about 0.5 for the quite narrow range of $\chi ^2$ values produced by the random PDF sets, and hence the effect of this on the final reweighted PDFs is very small. However, in practice there is surprisingly little variation with even lower values of $m$, and in terms of results simply ensuring that the function does not turn over appears to be sufficient. This insensitivity is unlikely to be so marked if a wider range of $\chi ^2$ values is produced, i.e. if the new dataset strongly constrains some eigenvector directions.

**Fig. 6:**
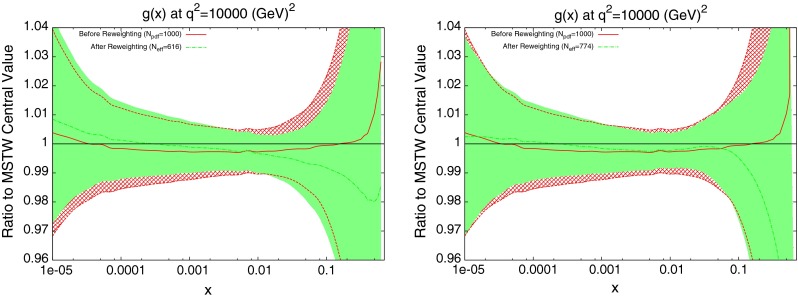
The effect of reweighting the MSTW2008 gluon using ATLAS inclusive jet data. Jet size parameter $R=0.4$ (*left*), and $R=0.6$ (*right*)

A number which can provide more information on the reweighting procedure is $N_\mathrm{eff}$, the effective number of PDFs included in the reweighted distribution. This is calculated by

6 $$\begin{aligned} N_\mathrm{eff}=\mathrm{exp}\left( \frac{1}{N_\mathrm{pdf}}\sum _{i=1}^{N_\mathrm{pdf}}w_i\ln \left( \frac{N_\mathrm{pdf}}{w_i}\right) \right) . \end{aligned}$$

If the dataset reweighted too has no effect, then all weights are 1 and $N_\mathrm{eff}=N_\mathrm{pdf}$, however, as soon as there are some weights larger than others, $N_\mathrm{eff}$ will provide an estimate for the number of random PDFs which have contributed.

The results of the reweighting procedure using $\mu = p_T$ are shown in Fig. 6 for the gluon, which is the only PDF noticeably affected by the data. There is a very slight trend for the gluon to increase at low $x$ and decrease at high $x$, but again it is clear that very little can be deduced with the swamping effect of the systematics. The reweighted PDF produces a comparison to data with a $\chi ^2$ of 0.73, slightly down from an unweighted value of 0.78.

Another issue regarding the treatment of systematics is that of whether to use multiplicative or additive definitions. The systematic errors in the data are presented as percentages, and so in order to obtain an absolute value of any given error, this percentage can be multiplied either by the data values or theory. If the percentage errors are multiplied by the data, they are considered additive since they are equivalent to an absolute error, whereas if they are multiplied by the theory they are considered multiplicative. By the nature of this particular fitting method, the data points themselves are significantly shifted in one direction by the systematics before the $\chi ^2$ is evaluated (in this case upwards, since the theory lies above data in general). Therefore, if the absolute errors are obtained from the raw data, they will be proportionally smaller after the shift. The effect of this can be seen in Table 3 where the $\chi ^2$ for the two separate treatments of errors is summarised. The multiplicative treatment shows a considerably lower $\chi ^2$ than the additive treatment, due to the larger absolute size of each error. The table also demonstrates that the physics being probed depends upon the treatment of the errors. In the multiplicative case with $R=0.6$, the best fit is obtained with a scale choice of $p_T$, whereas it is $2p_T$ when using additive. Whilst it is a small discrepancy, it shows the importance of the treatment of errors, since everything else in the two fits is identical.

**Table 3:** $\chi ^2$ per point using multiplicative and additive errors

Scale	$p_T$/2	$p_T$	2$p_T$
Multiplicative ($R=0.4$)	0.645	0.584	0.556
Multiplicative ($R=0.6$)	0.630	0.584	0.587
Additive ($R=0.4$)	0.752	0.773	0.703
Additive ($R=0.6$)	0.845	0.790	0.721

### ATLAS 2.76 and 7 TeV combined datasets

**Fig. 7:**
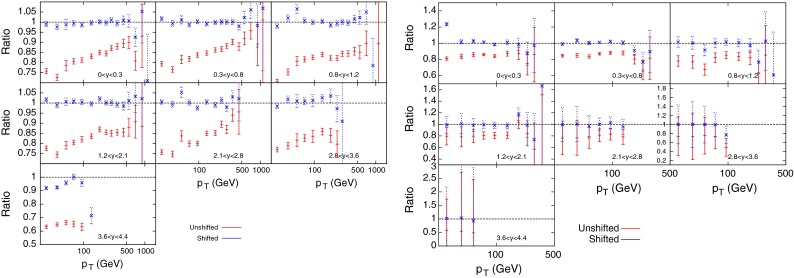
Ratio of data over theory for MSTW PDFs convoluted with APPLgrid for the ATLAS inclusive jet combined data. The left hand plots are the 7 TeV data points, whilst the right hand side shows the 2.76 TeV data. There is more fluctuation in the shifted points for 7 TeV with the constraints imposed by concurrent 2.76 TeV fit, than for the pure 7 TeV fit

A method for possibly reducing the effect of the systematic uncertainties of the inclusive jet cross section data is to perform a simultaneous fit of data taken at two different centre of mass energies, as done in [27]. The largest source of such uncertainties is the Jet Energy Scale (JES), which for ATLAS comprises 14 separate uncertainties correlated across all bins in the measurement. Since the source of JES uncertainties is the same at any centre of mass energy, performing a PDF fit across two measurements will significantly reduce the allowed systematic shift of data points, allowing better constraints on PDFs. The prediction for MSTW2008, using NLOjet++ interfaced with APPLgrid [28] is shown in Fig. 7, both before and after the systematics shifts in the $\chi ^2$ calculation are taken into account. The data again must be moved upwards for all points in the combined set to match the theory, however, when compared to the equivalent plot for the 7 TeV data (Fig. 3), it can be seen that the systematics are having less of an effect on this particular dataset, with more fluctuations in the shifted points, especially at high rapidity. Both the measurement of the inclusive jet cross section at 2.76 TeV and that at 7 TeV contain 21 sources of correlated systematic uncertainty which translate into 88 individual uncertainties after considering the correlations between rapidity bins. Only three of the sources are not correlated between the two datasets, and so the combined measurement contains 91 separate correlated uncertainties, an increase of only three, whilst increasing the data points from 90 to 149.

The original paper [27] to produce such a PDF analysis was produced by the HERAPDF Collaboration in conjunction with ATLAS. In this analysis, a minimum $p_T$ cut is applied of 45 GeV for all bins in both datasets, whilst the 2.76 TeV dataset includes a further maximum $p_T$ cut of 400 GeV which is applied in all but the $1.2<y<2.1$ rapidity bin. These cuts are motivated by the large hadronisation corrections in the stated bins, which can be as high as 12 % for some low-$p_T$ bins using $R=0.4$ (and higher using $R=0.6$). For this analysis, both definitions will be tested. The difference in fit quality is shown in Table 4, where a large improvement is seen when including the $p_T$ cuts for various PDFs. The source of this improvement is from the low-$p_T$ bins, where the statistical errors are the smallest, and so any deviation from the data produces a comparatively large increase in $\chi ^2$. Indeed, the source of the increase in $\chi ^2$ for the data without the cuts can be traced to one or two points in the set. The lowest-$p_T$ bin in the $0<y<0.3$ rapidity bin of the $2.76$ TeV dataset contributes over 100 points to the total $\chi ^2$ when using the MSTW2008 PDF set.

**Table 4:** $\chi ^2$ per point for ATLAS combined data, both with and without $p_T$ cuts. The third column uses additive errors and has two additional anomalous points cut

	No cuts	HERAPDF cuts	Additive errors
MSTW 2008	1.43	0.93	1.46

As discussed for the pure 7 TeV fit, the way in which the systematic errors are treated is important to the quality of fit due to the systematic shift between data and theory. So far for this combined dataset the multiplicative definition has been used since this is the treatment which most closely follows the HERAPDF/ATLAS analysis. Now, the additive definition is discussed. Since the same shift upwards from the data to the theory is seen in the ATLAS combined dataset, it is expected to give a worse fit. This is true, and for MSTW2008 NLO PDFs, the fit becomes 2.44 per point, more than doubling the $\chi ^2$ from the multiplicative treatment. However, a large part of this $\chi ^2$ is localised to two anomalous points, even after the HERAPDF cuts. These are the highest-$p_T$ bin of the highest rapidity bin of the 7 TeV data, and the lowest-$p_T$ bin (after cuts) of the third rapidity bin of the 2.76 TeV data. Removing just these two additional points reduces the $\chi ^2$ to 1.46 per point (the effect is rather less pronounced for the $R=0.6$ data). Since the MSTW fitting code currently uses additive errors for all datasets, it is proposed to remove these points for a PDF fit including this data. The $\chi ^2$ value is shown in the third column of Table 4.

**Fig. 8:**
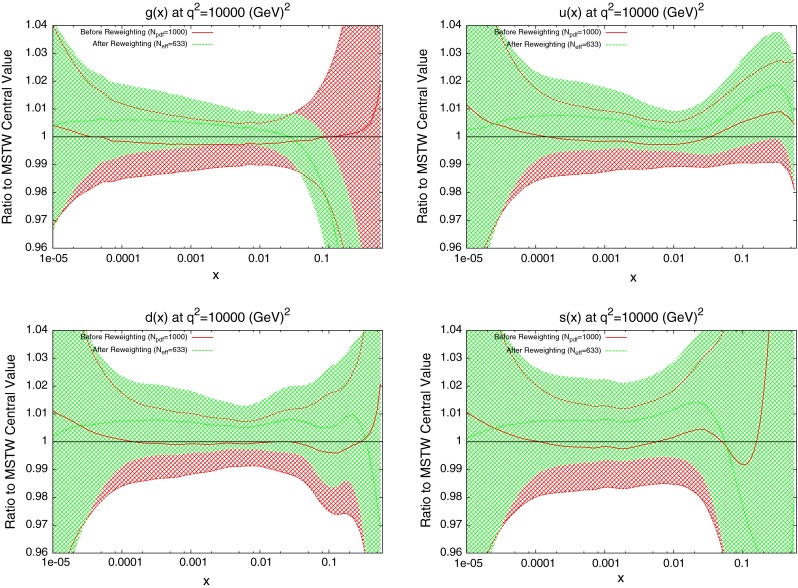
Effect of the ATLAS combined inclusive jet data on the gluon and quark PDFs. Here, multiplicative errors are used, and the lowest two bins in $p_T$ in all rapidity bins and the highest-$p_T$ bins in the 2.76 TeV rapidity bins are excluded as per the HERAPDF analysis

**Fig. 9:**
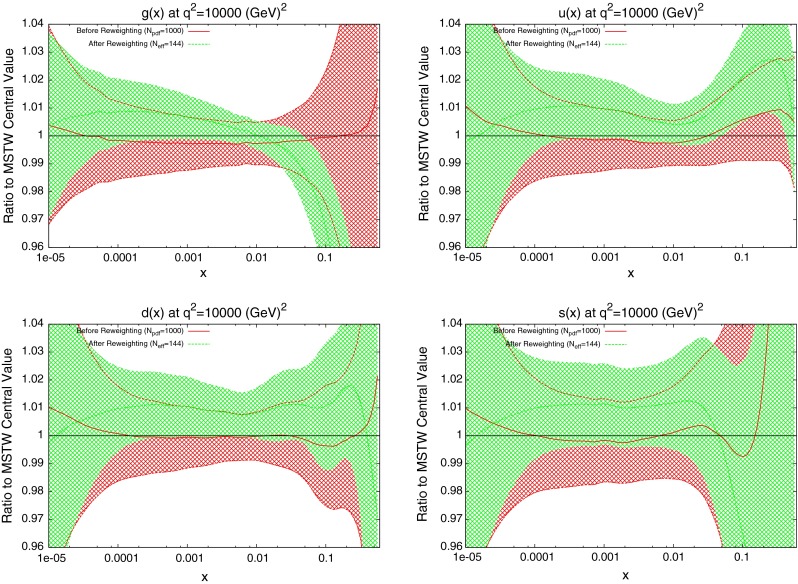
Effect of the ATLAS combined inclusive jet data on the gluon and quark PDFs. Here, additive errors are used in the determination of the $\chi ^2$, and the two anomalous points discussed in the previous section are additionally cut

The effect on the PDFs using the reweighting technique is shown for the case of multiplicative errors in Fig. 8 and for additive errors in Fig. 9. In both cases, the central value of the reweighted gluon is consistent with the standard MSTW 2008 central value across all values of $x$ and in the multiplicative case it is very similar to the reweighted pure 7 TeV gluon. The error bands are reduced in size more significantly than when using just the 7 TeV data, and the additive treatment seems to have more of an effect in this sense than the multiplicative. The upward shift in the quark PDFs and also the error constraints are larger when using the additive treatment. Clearly there is more constraint on the gluon with the 2.76 TeV data included, and the reduction in systematics is allowing more information on the PDFs to be extracted. The quark PDFs are also shown; although the effect is again larger than the pure 7 TeV case, there is very little movement from the central MSTW value. When using multiplicative errors, the $\chi ^2$ is reduced from 0.974 to 0.962 by reweighting and $N_\mathrm{eff}=633$, and for additive errors the effect is larger as expected from the reweighted plots, changing from 1.45 to 1.26, and $N_\mathrm{eff}=144$, a much lower value. (Note that the $\chi ^2$ obtained from the average of the random PDF sets is not identical to that obtained using the best fit PDF set, but it is always very close.) Whilst the two additional data points cut were deemed necessary to provide an acceptable $\chi ^2$ value, it was observed that even when including these points, the reweighed PDFs for the additive treatment were essentially unchanged. Hence, the difference between Figs. 8 and 9 can be attributed to the differing error treatments.

### CMS inclusive jets

**Fig. 10:**
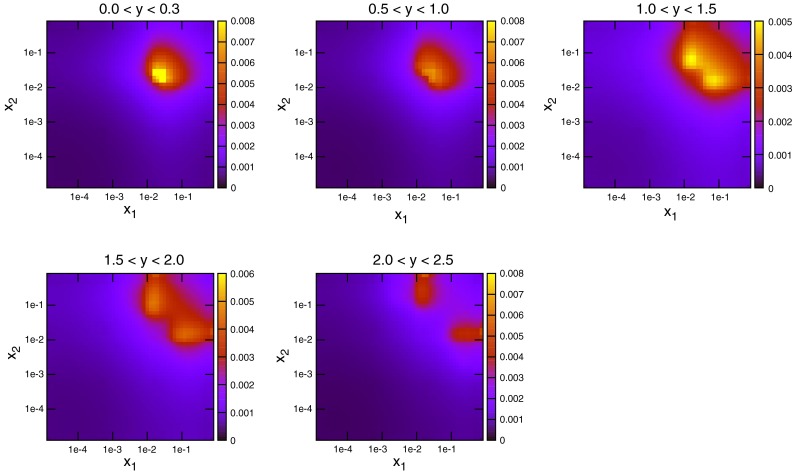
Distribution of $x_{1,2}$ values for NLOjet++ events in the CMS inclusive jet calculation

**Fig. 11:**
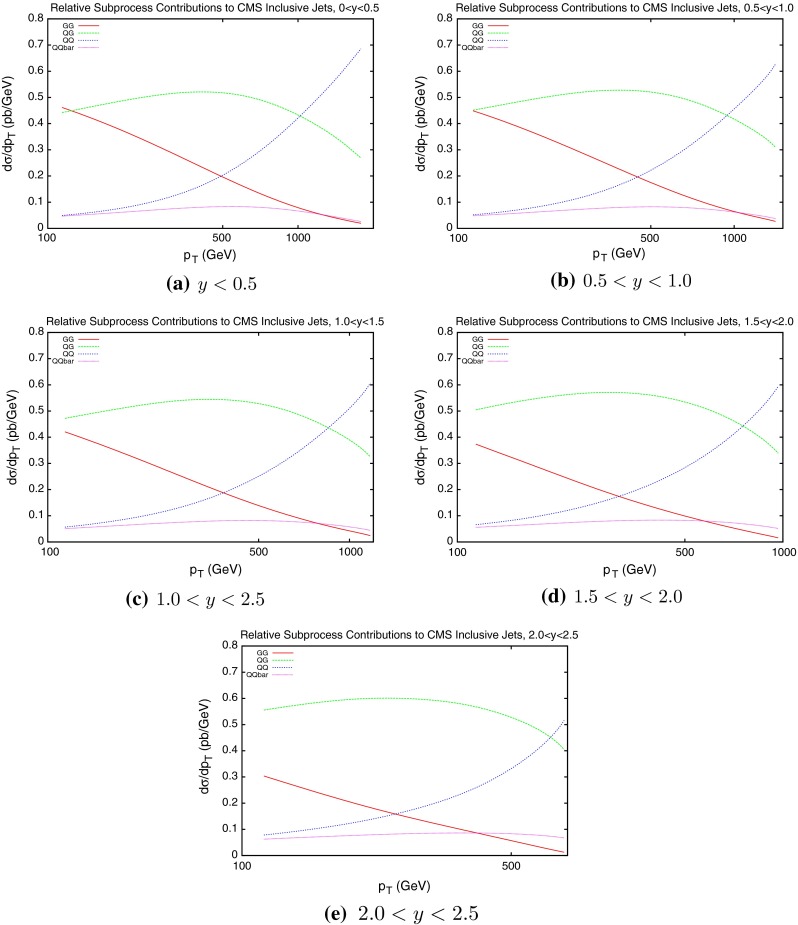
Contributions of different initial-state parton combinations to the CMS inclusive jet cross section calculation

To date the LHC dataset with the most resolving power for PDFs is that released by the CMS Collaboration [29] in early 2013. This analysis, like the earlier ATLAS analysis, was performed at 7 TeV. However, a much higher collected luminosity of 5 fb$^{-1}$ is included, and so statistical errors are greatly reduced across the phase space. Compared to the ATLAS measurement, the jet $p_T$ spectrum extends much higher to 2 TeV, whereas it is also cut off higher, only going down to 114 GeV. There is also less rapidity span for the CMS jets, which are only measured to a rapidity of 2.5. The overall effect is to have more pronounced sensitivity to high-*x* PDFs, and lower sensitivity to low-*x* PDFs. This can be seen in Fig. 10, where the $(x_1,x_2)$ distribution for each event generated is shown. The reach to low $x$ is limited to $10^{-3}$, but each distribution is shifted more towards the high $(x_1,x_2)$ region. The partons which are probed by the data are therefore naturally different from those of ATLAS. The greater emphasis on medium- to high-$x$ partons means a greater relative contribution from quarks. Figure 11 shows the partonic composition of the calculation at each point in phase space. Unlike the ATLAS jets, the $gg$ subprocess does not dominate anywhere in the phase space, with $gq$ contributing maximally everywhere except for the very highest-$p_T$ jets.

**Fig. 12:**
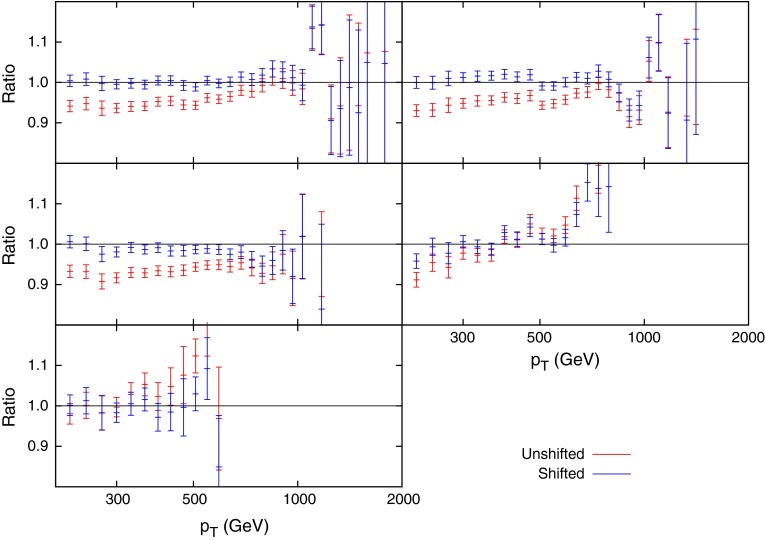
Ratio of data to theory using MSTW 2008 NLO for CMS inclusive jets. Both the raw APPLgrid calculation and the calculation after systematic effects are taken into account are shown

The raw calculation using NLOjet++ interfaced with APPLgrid is in much better agreement with data than the ATLAS inclusive cross section. Whilst the ATLAS jet calculation was up to 30 % too high in some bins, the CMS calculation is never more than 10 % off. The systematics must again be taken account of in a $\chi ^2$ fit, and the comparison to data again improves after this consideration. However, as Fig. 12 shows, the shifted data/theory points reflect the statistical fluctuations present in the unshifted points. For the ATLAS fit, it was clear that the statistical fluctuations were being washed out by the large freedom provided by the systematics. The table of fits is shown in Table 5, and the corresponding systematic shifts in Table 6. The $\chi ^2$ values are generally worse than for the ATLAS data. With fewer $r_k$ values, it is clear that there is less freedom to compensate for differences by using the systematic shifts. This is reflected by the distribution of the $r_k$, which for ATLAS produced a majority below 0.5, but for CMS it has a larger number of higher values.

**Table 5:** $\chi ^2$ per point (133 points) for NLO PDFs for CMS inclusive jet data

Scale	$p_T$/2	$p_T$	2$p_T$
MSTW 2008	1.92	1.48	1.12

**Table 6:** Distribution of the $r_k$ (total 19)

$|r_k|$	${<}0.5$	0.5–1.5	1.5–2.5	2.5–3.5	3.5–4.5
MSTW 2008	8	8	2	1	0

**Fig. 13:**
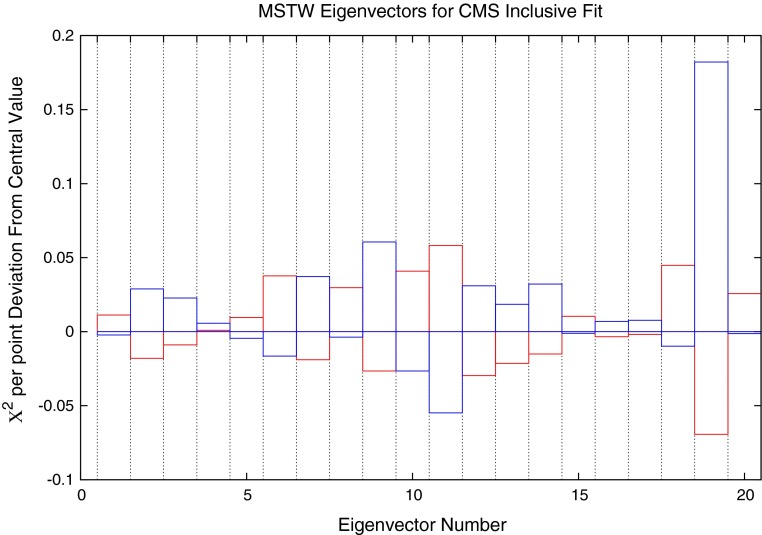
Change in fit quality from the central MSTW2008 PDF for each eigenvector in the set

The same procedure as described for the ATLAS jets is applied to the CMS dataset. The variations of the fit under movements in the eigenvector directions are shown in Fig. 13. This time, there are significant improvements in some directions, with eigenvectors 11 and 19 reducing the $\chi ^2$ the most. Eigenvectors 19 is most influenced by the gluon distribution while 11 contributes significantly to the uncertainty of a wide variety of PDFs.

**Fig. 14:**
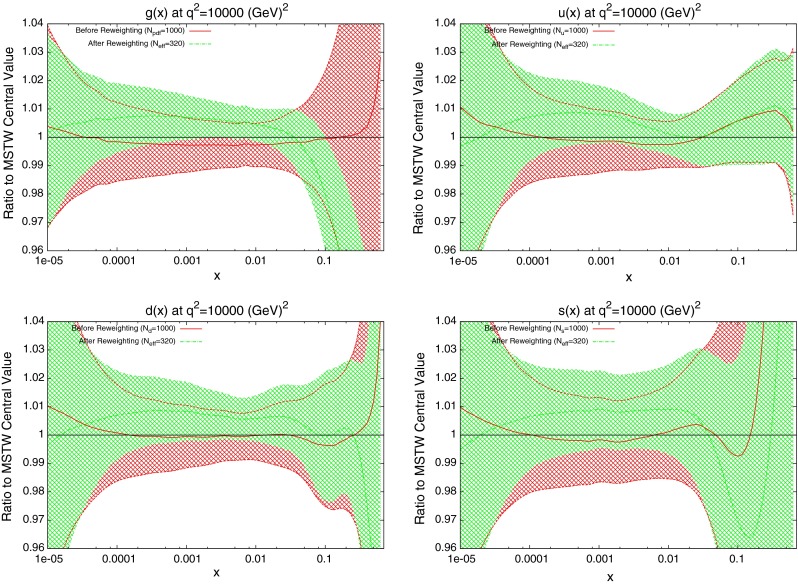
Effect of the CMS inclusive jet data on the gluon and quark PDFs

When the reweighting procedure is applied, the results of which are shown in Fig. 14, the effect is larger than the full ATLAS combined dataset. The shape of the reweighted gluon agrees with the ATLAS reweighting, with a lower gluon at high $x$. What is significant in this case is the increased sensitivity to the quark PDFs. The reduction in error band in the up and down distributions is similar to that for the gluon. Even the error in the strange distribution is reduced in both directions across almost all values of $x$. The focus of the CMS data on higher values of $x$ has lead to a less dramatic effect on the gluon, but consistently better constraining of all quark PDFs. The reweighting improves the fit quality from 1.47 to 1.29.

**Fig. 15:**
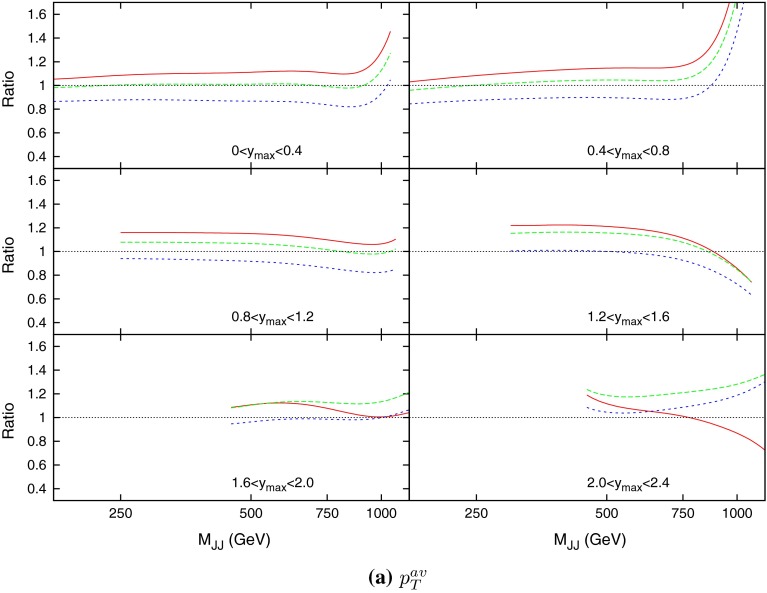
Theory/data ratio for DØ dijets, using multiples of $p_T^{av}$ as the choice of $\mu _R$ and $\mu _F$. The multiples are $0.5$ (*red*), $1.0$ (*green*) and $2.0$ (*blue*)

## Dijet cross sections

In all previous MSTW fits, only inclusive jet data has been included into the fit. This is due to the overlap with the inclusive jet cross sections, but also to the uncertainties in the calculation of dijet cross sections and the scale choices therein. Whilst there is very limited scope for changing the kinematic choice of scale for inclusive jets, there are many possibilities when considering dijet cross sections. As such, this section presents a thorough study of the effect of the choice of renormalisation and factorisation scale choice on dijet predictions at both the Tevatron and the LHC, and the feasibility of including these datasets in a PDF fit is tested.

Before 2011, the only dijet data available over a range of rapidity was from DØ at the Tevatron. Studies into the comparison between data and theory were conducted [8], but inconsistencies in the scale uncertainty were found. The NLO calculations for the dataset were performed using the average jet $p_T$ as the scale choice, and this was shown to exhibit strange behaviour at high rapidities. This is demonstrated in Fig. 15, where the predictions over (smoothed) data for 0.5, 1 and 2 times the scale choice are shown to cross over at high $y_\mathrm{max}$ and mass, and for understand the source of this behaviour, the kinematics of the process must be studied.

### Kinematics of dijet production

**Fig. 16:**
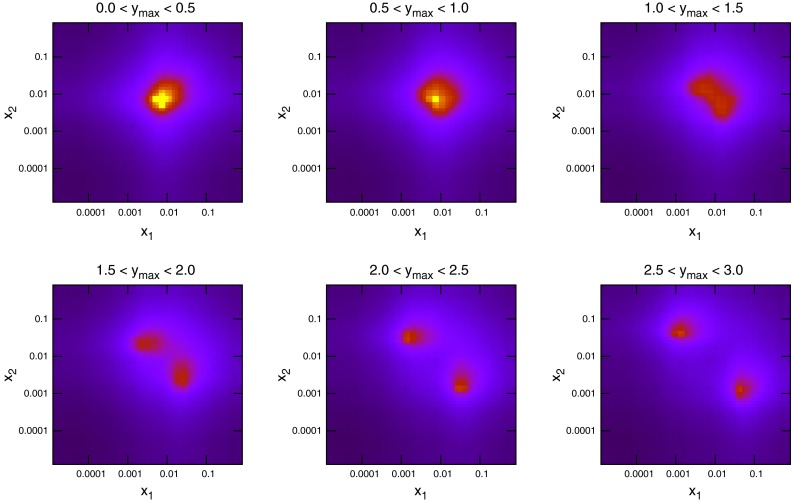
Values of $x_1$ and $x_2$ for each event generated in NLOJet++ for dijets at DØ

**Fig. 17:**
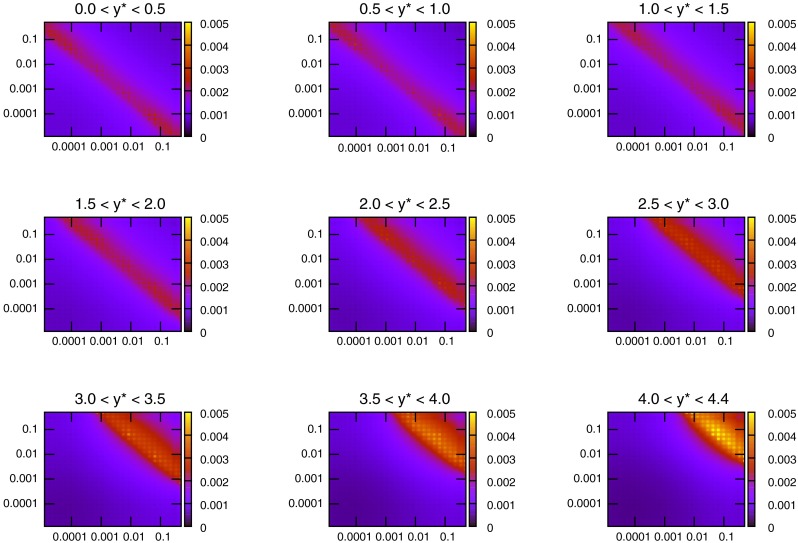
Values of $x_1$ and $x_2$ for each event generated in NLOJet++ for dijets at ATLAS

The kinematics of the dijet production process are defined using the invariant mass of the dijet system, $M_\mathrm{JJ}$, and the rapidity of each of the jets in the event. A double-differential cross section is constructed using bins in the dijet mass and a combination of the two rapidities. The flexibility in the latter leads to different possibilities for rapidity binning, and the DØ and ATLAS [15] measurements use differing definitions. Where DØ uses $y_\mathrm{max}$, the maximum rapidity of the two jets comprising the dijet pair, ATLAS chooses $y^*=(y_1-y_2)/2$, the difference between them. This is the cause of the greatly differing $x$ distributions of Figs. 16 and 17. Using the maximum jet rapidity results in a similar pattern to inclusive jets, due to the fact that only the rapidity of one jet is considered. At high rapidities a single high-$x$ parton must combine with a single low-$x$ one, and low rapidities require equal values of $x$ in both partons. Using the rapidity difference,

however, allows a much wider range of parton momentum fractions to produce dijets in all $y^*$ bins. The observed shift towards high $x$ at high $y^*$ is in fact due to the fact that only high-$M_\mathrm{JJ}$ events are measured at these rapidities. These high-$M_\mathrm{JJ}$ events are also present in the other rapidity bins, however, due to the power-like drop in cross section with dijet mass these events do not register in the respective plots and only the lowest-mass bins can be seen.

**Fig. 18:**
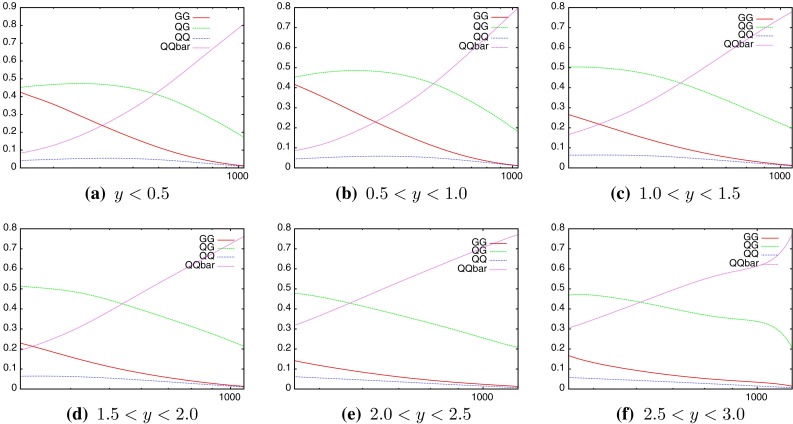
Contributions of different initial-state parton combinations to the DØ dijet cross section calculation

**Fig. 19:**
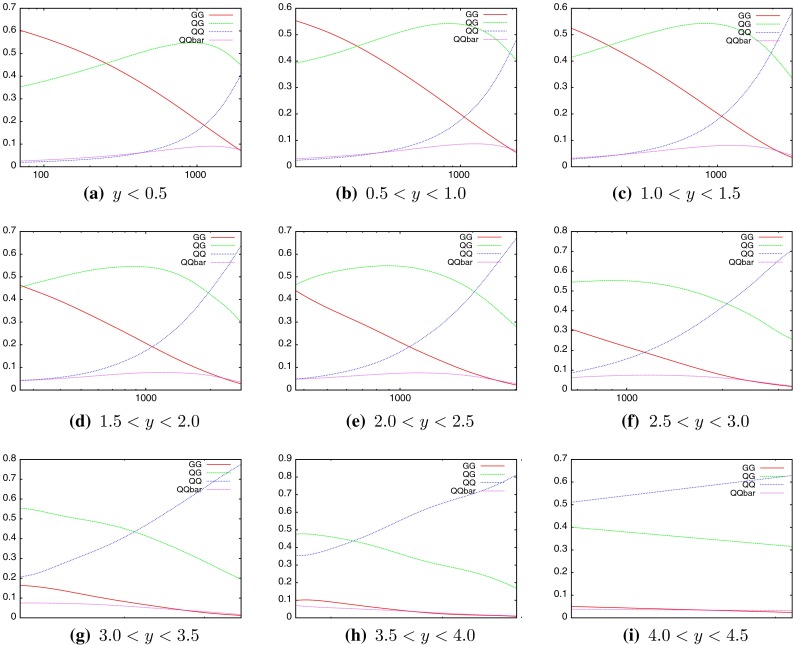
Contributions of different initial-state parton combinations to the ATLAS dijet cross section calculation

The difference in the distributions of parton momenta leads to the question of which partons are being probed at different points in the phase space. Here we can begin to see the differences in the various datasets, especially when comparing to the relevant commensurate inclusive jet data. For the DØ dijet cross section in Fig. 18, it is clear that the quark PDFs are in general the most important, with the $gg$ luminosity always below the $qg$, and mostly below the $q\bar{q}$ luminosities. For the ATLAS dijets, Fig. 19 shows that for low rapidities, a similar behaviour to the corresponding inclusive jet plot is seen, with the gluon density dominating until the very high-$M_\mathrm{JJ}$ region. However, at higher rapidities, the requirement of two high-$x$ partons means the $qq$ luminosity becomes by far the most important. As a result, the dijet data for ATLAS should affect the quark densities far more than when using only the inclusive data.

### Scale Variations

When considering dijet production, the choice of renormalisation and factorisation scales to include in the NLO calculation is not obvious. In general the behaviour of varying the scale on the full NLO calculation performed by NLOjet++ can be seen in the form of the differential cross section:

$$\begin{aligned} \frac{\mathrm{d}^2\sigma }{\mathrm{d}M_\mathrm{JJ}\mathrm{d}y}\!&=\left[ \alpha _s^2(\mu _R)\sigma _\mathrm{LO}+\alpha _s^3(\mu _R)\left( \sigma _\mathrm{NLO}\right. \right. \nonumber \\&\quad \left. \left. \!+2b_0\log \left( \frac{\mu _R}{M_\mathrm{JJ}}\!\right) \!\sigma _\mathrm{LO}\!-\!2\log \!\left( \frac{\mu _F}{M_\mathrm{JJ}}\right) \!P_{ab}\otimes \sigma _\mathrm{LO}\!\right) \!\right] \\&\quad \otimes f_a(\mu _F)\otimes f_b(\mu _F) \end{aligned}$$

where the leading order and next to leading order cross sections, $\sigma _\mathrm{LO}$ and $\sigma _\mathrm{NLO}$ are computed using the matrix elements and evaluated at $\mu _R=\mu _F=M_\mathrm{JJ}$, $b_0$ is the leading order QCD beta function coefficient, and $P_{ab}$ are the QCD splitting functions. The behaviour of this cross section under renormalisation scale variations is relatively simple, with only the running of $\alpha _s$ and a logarithm including this variable. The factorisation scale variations, however, are sensitive to the convolution with the PDFs, and so the particular $x$ values and partons probed in a particular event will affect the variations in $\mu _F$.

**Fig. 20:**
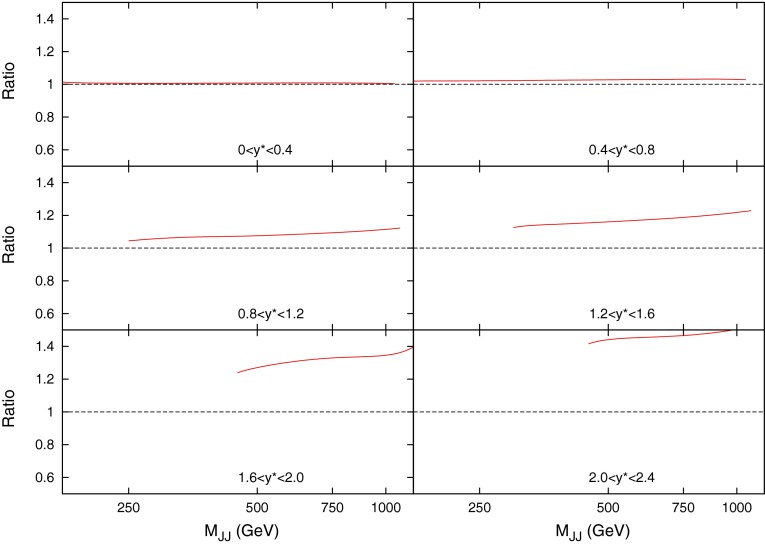
Ratio of $M_\mathrm{JJ}$ calculation to $2p_T^{av}$ calculation for DØ dijet calculation. The equivalence of the two scale choices at central rapidities is apparent, with large deviations for more forward jets. Both calculations are performed with NLOjet++

Unlike inclusive jet production, in which the only physical scale involved in the events is the $p_T$ of the jet, dijet production has a number of possible choices of scale. The seemingly most obvious choice is the average $p_T$ of the two jets, however, at high rapidities this can lead to problems due to the possible configuration of the event. A highly boosted hard scatter will have the same average $p_T$ as an unboosted soft scatter. Another variable which could be used as the scale choice is the dijet mass, $M_\mathrm{JJ}$. This does not suffer from the same issues in event classification at high rapidities, though in this case it is possible to have a very soft high-rapidity scatter which still has high $M_\mathrm{JJ}$. At leading order, the mass is defined as

7 $$\begin{aligned} M_\mathrm{JJ}=2p_T\cos { h}(y^*) \end{aligned}$$

where $y^*=(y_\mathrm{jet 1} - y_\mathrm{jet 2})/2$ is half the rapidity difference of the final state jets making the dijet pair. At the limit $y^*=0$, for fully back-to-back jets, we have $M_\mathrm{JJ}=2p_T$ as expected, and so the predictions using the two scale choices should agree. This is demonstrated in Fig. 20, where the dijet cross section is calculated using both scales, and the ratio shown.

**Fig. 21:**
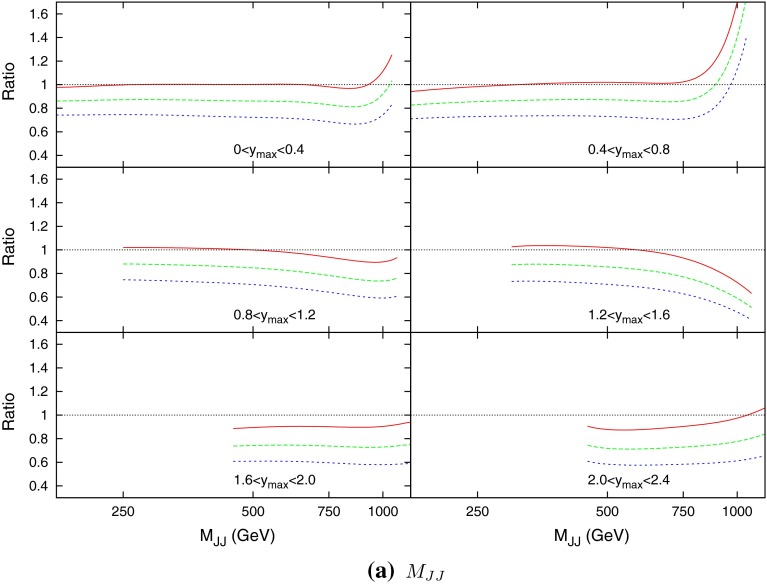
Theory/data ratio for DØ dijets, using multiples of $M_\mathrm{JJ}$ as the choice of $\mu _R$ and $\mu _F$. The multiples are $0.5$ (*red*), $1.0$ (*green*) and $2.0$ (*blue*)

Figure 21 (in comparison to the $p_T^{av}$ plot Fig. 15) demonstrates the apparent benefit of using dijet mass as the scale choice. In the case of $p_T^{av}$, although at low rapidity the prediction is stable and flat across all $M_\mathrm{JJ}$, the predictions from different multiplicative factors of the scale begin to cross in the more forward bins. This has already been observed in [8], however, other scale choices were not investigated. In comparison, the theory/data ratio for the $M_\mathrm{JJ}$ calculation is much more stable. The variation through multiplicative factors of the scale are constant throughout all rapidity bins, and the ratio generally remains flat.

The $\chi ^2$ values shown in Tables 7, 8 and 9 confirm that the choice of $M_\mathrm{JJ}$ provides the better fit to the DØ data. Also calculated is another choice of scale, namely multiples of $\frac{M_\mathrm{JJ}}{2\cos { h}(0.7y^*)}$. This form of scale choice was suggested [30] as an empirical means to stabilise NLO corrections, and it is almost equivalent to the choice $p_T\exp (0.3y^*)$ used by ATLAS [15]. This choice allows the dependence on the dijet rapidity to be directly included. While it also provides an improvement on the $p_T^{av}$ calculation, it does not provide as good a fit for the DØ dijets as using simply $M_\mathrm{JJ}$.

**Fig. 22:**
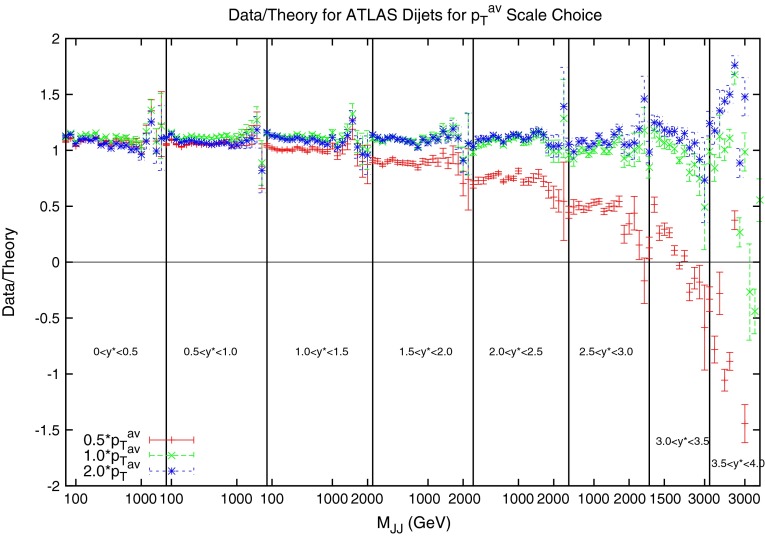
Ratio of data to theory for ATLAS dijets using three different multiples of $p_T^{av}$ as the scale choice. For the multiple of 1.0, the cross section becomes negative at high rapidity. This occurs much earlier for the lower multiple of 0.5

**Table 7:** $\chi ^2$ values for DØ dijets

	$0.5*p_T^{av}$	$1.0*p_T^{av}$	$2.0*p_T^{av}$
MSTW2008 NLO	3.23	2.34	1.61

**Table 8:** $\chi ^2$ values for DØ dijets

	$0.5*M_\mathrm{JJ}$	$1.0*M_\mathrm{JJ}$	$2.0*M_\mathrm{JJ}$
MSTW2008 NLO	1.88	1.29	1.06

**Table 9:** $\chi ^2$ values for DØ dijets

	$0.5*\frac{M_\mathrm{JJ}}{2\cos { h}(0.7y^*)}$	$1.0*\frac{M_\mathrm{JJ}}{2\cos { h}(0.7y^*)}$	$2.0*\frac{M_\mathrm{JJ}}{2\cos { h}(0.7y^*)}$
MSTW2008 NLO	3.06	2.15	1.44

The equivalent ATLAS results are now shown in Tables 10, 11 and 12. The tendency for the $p_T^{av}$ calculation to degrade at small multiplying factors is even more apparent here than with the DØ dijets, so much so that the $0.5p_T^{av}$ is not shown, and all values are multiplied by a further factor of 2. Even with this additional factor, the $1*p_T^{av}$ fit is terrible, and is due to the cross section calculation being negative in the high-rapidity, high-mass region, which can be seen in Fig. 22. This plot clarifies the issue with using $p_T^{av}$, which initially appeared in the DØ calculation, since it includes much higher rapidity and mass regions. It is clear that as higher rapidities are reached, the $p_T^{av}$ calculation dramatically falls off for low multiplying factors, to the point where it becomes negative for both the 0.5 and the 1.0 factors. Despite this, once the multiplying factor is large enough, $p_T^{av}$ provides the best fit of the three choices, with $M_\mathrm{JJ}$ in fact showing the worst fit of the three.

**Table 10:** $\chi ^2$ values for ATLAS dijets

	$p_T^{av}$	$2.0*p_T^{av}$	$4.0*p_T^{av}$
MSTW2008 NLO	6.66	1.94	1.91

**Table 11:** $\chi ^2$ values for ATLAS dijets

	$0.5*M_\mathrm{JJ}$	$1.0*M_\mathrm{JJ}$	$2.0*M_\mathrm{JJ}$
MSTW2008 NLO	2.09	2.43	3.00

**Table 12:** $\chi ^2$ values for ATLAS dijets

	$0.5*\frac{M_\mathrm{JJ}}{2\cos { h}(0.7y^*)}$	$1.0*\frac{M_\mathrm{JJ}}{2\cos { h}(0.7y^*)}$	2.0*$\frac{M_\mathrm{JJ}}{2\cos { h}(0.7y^*)}$
MSTW2008 NLO	2.59	2.27	2.11

**Fig. 23:**
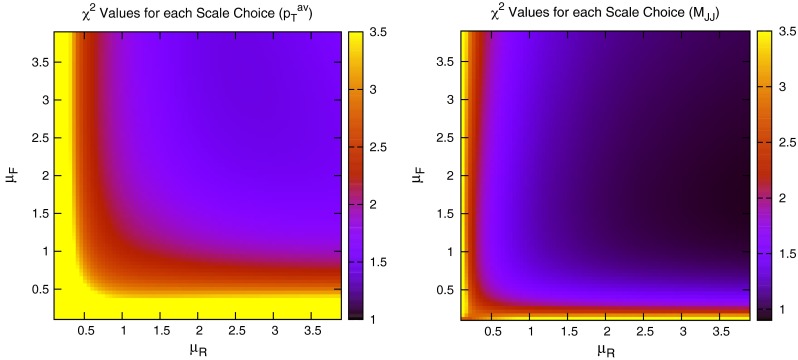
$\chi ^2$ per point for all values of multiplication factor for both $p_T^{av}$ and $M_\mathrm{JJ}$ calculations for DØ dijets. The *yellow area* at low scales in the $p_T^{av}$ calculation is greatly off the scale, due to the calculation becoming negative in this region

**Fig. 24:**
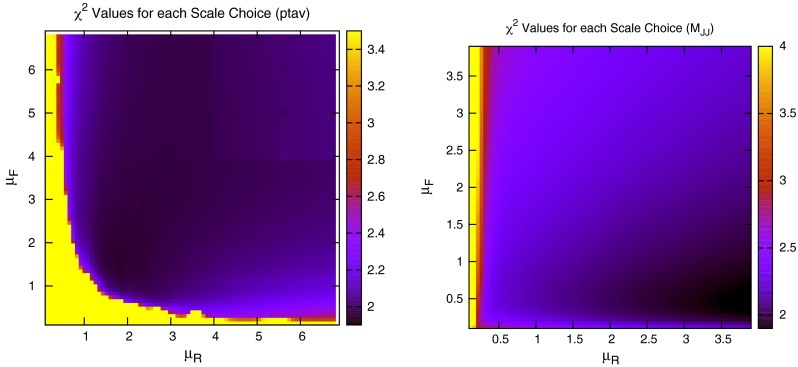
$\chi ^2$ per point for all values of multiplication factor for both $p_T^{av}$ and $M_\mathrm{JJ}$ calculations. For ATLAS dijets the *yellow area* at low scales in the $p_T^{av}$ calculation is greatly off the scale, due to the calculation becoming negative in this region

When considering the entire space of fits for any combination of $(\mu _R,\mu _F)$, using the dijet mass is again shown to be a more stable prediction that average $p_T$. Figures 23 and 24, which show the fit quality for DØ and ATLAS, respectively, more completely shows the degradation of the $p_T^{av}$ calculation at low values of scales. The yellow region, which for DØ covers the area in which either scale is below $0.5$, shows a rapid unbounded increase in $\chi ^2$, deriving from the fact that the cross section becomes increasingly negative as the scales approach 0. The fit becomes comparable in quality to the $M_\mathrm{JJ}$ calculation at much higher choices of scale, however, there is no clear minimum which can be identified as a stable choice. For ATLAS, the region of divergent $\chi ^2$ is much larger for $p_T^{av}$, with normally sensible choices showing a very poor fit. Again, this is the result of the larger kinematic span of the ATLAS dijets exposing the failure of this calculation in the high-rapidity, high-mass region.

The $M_\mathrm{JJ}$ calculation for both datasets shows a similar trend by increasing towards lower scale choices. However, due to the stability at high rapidities, the fit does not blow up in the same way as for $p_T^{av}$, and a lower $\chi ^2$ is apparent across the entire parameter space. There is a much clearer minimum, although it occurs for unusually high values of $\mu _R$. This issue is discussed later and is shown to be related to the normalisation uncertainty.

**Fig. 25:**
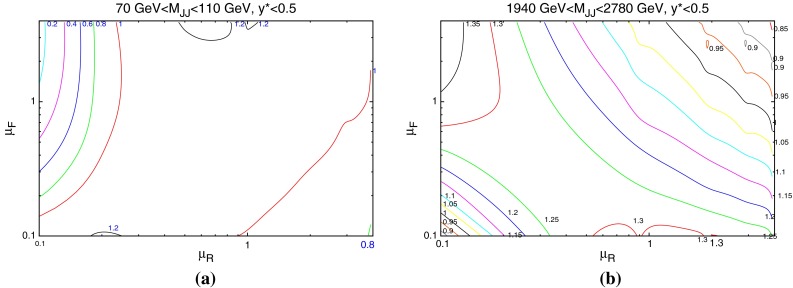
Comparison of scale variations for the **a** lowest- and **b** highest-$M_\mathrm{JJ}$ bins in the $y^*<0.5$ rapidity bin of the ATLAS dijet calculation. The contour values are data/theory. The scales $\mu _R$ and $\mu _F$ are multiples of $M_\mathrm{JJ}$

**Fig. 26:**
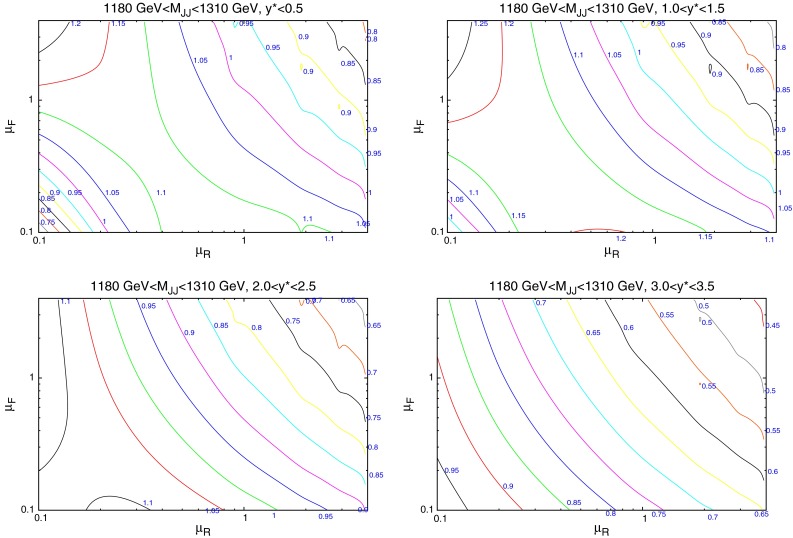
Comparison of scale variations for different rapidity bins. The same $M_\mathrm{JJ}$ range is used throughout

The nature of the effect of scales can be more deeply probed by studying individual cross sections in finely defined regions of phase space. Whereas the previous discussion has focussed on the fit to data of an entire dataset, the following will study the variation of each point within that dataset for each scale choice. As Figs. 18 and 19 have shown, the contributions from the individual PDFs depends greatly on the values of the kinematic variables, and so the variation of each point in the factorisation scale direction should change in a similar manner. Figure 25 (similar to plots in [31]) demonstrates the scale variation of two single points in the kinematic phase space of the ATLAS dataset. Both are in the lowest-$y^*$ bin, however, the first includes dijets with low mass (70–110 GeV) and the second includes those with high mass (1,940–2,780 GeV). The general behaviour is that of a stable saddle region in the central region, with data/theory decreasing away from the saddle along one axis and increasing along the other. The axes defining the saddle region, however, differ greatly between the two points. A smooth rotation anticlockwise is observed as the dijet mass is increased, resulting in the large rotation shown in the figure. The dependence of this rotation on the kinematic variables is shown more clearly in Fig. 26, where only the rapidity bin is changed. The $1.18~\mathrm{TeV}<M_\mathrm{JJ}<1.31~\mathrm{TeV}$ bin is chosen for study as this is the bin appearing in the most rapidity bins. It is clear that the angle of the saddle point is dependent only on the dijet mass, however, the overall behaviour is still affected by the rapidity. A migration towards lower scale choices is seen, such that at the highest rapidities, the saddle point disappears and the surface simply becomes a unidirectional slope. Ideally, the scale choice for a calculation would be the one which provides the most stable calculation, and hence would be within the saddle region for all of the points in the dataset.

**Fig. 27:**
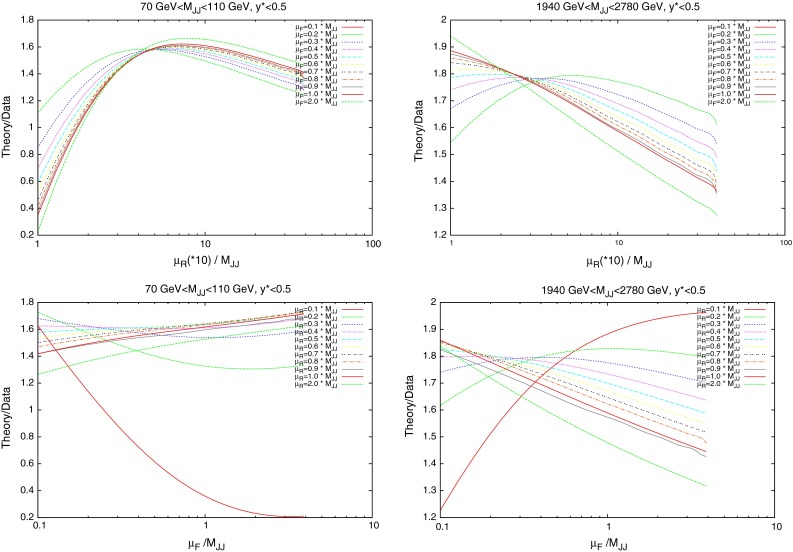
Plots demonstrating the variation of $\mu _R$ and $\mu _F$ independently

In order to understand the source of the observed behaviour, the variation in $\mu _R$ and $\mu _F$ are studied independently. Figure 27 demonstrates this for two points, at low and high $M_\mathrm{JJ}$ for the scale choice of $M_\mathrm{JJ}$. The observed behaviour demonstrates that the rotation as a function of the dijet mass is governed by the factorisation scale changes. The renormalisation scale changes are similar at all values of $M_\mathrm{JJ}$, with a smooth shape that changes little as the slices move through the factorisation scale range. The $\mu _F$ dependence, however, changes greatly with the dijet mass. In the first plot, with the lowest-$M_\mathrm{JJ}$ bin in the lowest-rapidity bin shown, the factorisation scale dependence is roughly flat for all slices in $\mu _R$ except for the very lowest two $\mu _R$ choices. This is the cause of the vertical nature of the saddle point in the first plot in Fig. 25. In the second plot, at high $M_\mathrm{JJ}$ in the lowest-rapidity bin, the factorisation scale has a non-flat shape that depends greatly on the value of $\mu _R$ chosen. Because the variations in factorisation scale are now large, the saddle point in the second plot in Fig. 25 is no longer vertical, and is rotated anticlockwise. The higher $\mu _F$ dependence at high $M_\mathrm{JJ}$ can be understood through the $x$ values probed. In the high-$M_\mathrm{JJ}$ region, the high-$x$ partons necessary for the events are evolved much more quickly than at medium/low $x$, and so a greater dependence on the factorisation scale is seen. The stability of the calculation, then, is dependent on the partons probed.

**Fig. 28:**
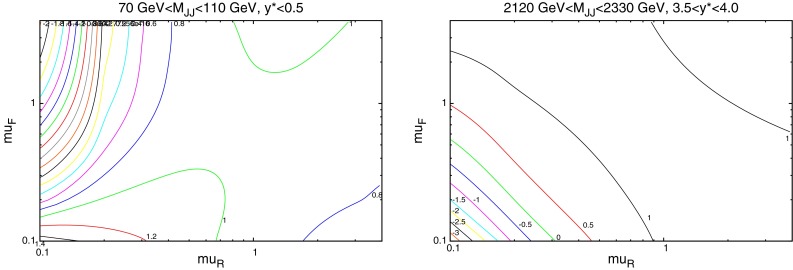
Scale variations for the scale choice $M_\mathrm{JJ}/2\cos { h}(0.7y^*)$. Unlike when using $M_\mathrm{JJ}$, the saddle point remains centrally located even in the high-rapidity region

The need to choose a single scale for the entire calculation leads to the search for a choice where the saddle point is uniformly based at that choice. Since the calculation using $M_\mathrm{JJ}$ as the kinematic scale choice seems to fail at higher rapidities, a function of $M_\mathrm{JJ}$ and $y^*$ would be a logical choice to attempt to modulate this behaviour. The function $M_\mathrm{JJ}/2\cos { h}(0.7y^*)$ is studied, which was shown in the previous section to improve the stability of the ATLAS calculation. The scale variations for this choice are shown in Fig. 28, where even in the highest-rapidity bin, the saddle point is located around the central scale choice. It is clear that for the ATLAS dataset, the phase space probed would prefer a scale choice including a rapidity term.

**Fig. 29:**
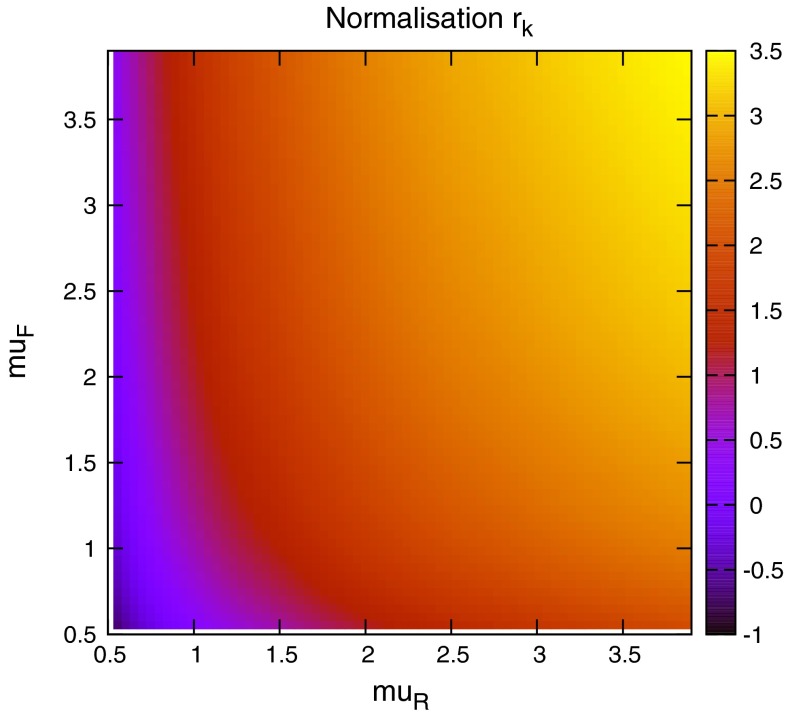
Value of the systematic shift associated with the normalisation uncertainty for each scale value (multiples of $M_\mathrm{JJ}$)

### Data normalisation

The treatment of normalisation errors on datasets has been a subject of previous discussion [9], and it is important to understand the effect they have on a fit. The only experimental source of the error is the luminosity uncertainty of the collider, and so it is correlated across all datasets produced at a single collider. For the Tevatron Run II data, the luminosity uncertainty is $6.1~\%$, whilst the ATLAS 7 TeV run has a $3.4~\%$ error. These provide the possibility for a theoretical prediction to move greatly up or down whilst incurring only a small penalty term in the $\chi ^2$. Due to this effect, the MSTW 2008 PDFs include a more severe quartic penalty term for the normalisation.

**Fig. 30:**
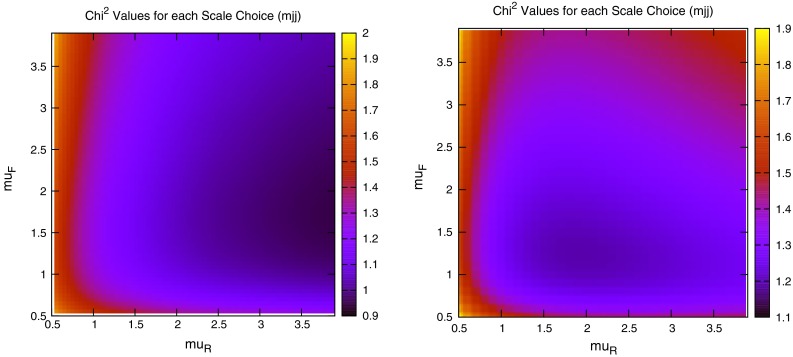
Goodness of fit for each combination of scales (multiples of $M_\mathrm{JJ}$), first with and second without allowing the normalisation to move freely

When considering the best choice of scale variable for *DØ* dijets, namely $M_\mathrm{JJ}$, the best possible fit is obtained at very high values of renormalisation scale, as represented in Fig. 30. However, if the normalisation $r_k$ of each fit is studied (where positive values of $r_k$ mean the data is normalised down), it is clear that this minimum is obtained in a region where a $~2$–$3 \sigma$ shift is required, as can be seen in Fig. 29. In fact, there is a very small area of the parameter space in which the normalisation parameter is moved less than $1 \sigma$, though this does include $\mu _{R,F}=M_\mathrm{JJ}$. The second plot in Fig. 30 represents the same fit, but keeping the normalisation fixed. The minimum is now at a more sensible scale choice, at the cost of requiring a slightly higher $\chi ^2$. Clearly equation 1 is inadequate for providing the most sensible fit, and a different treatment of the normalisation $r_k$ will ultimately be required. The difference in the normalisation treatments is most important for high values of the scales, where the calculation would naively appear to give the best fit. The effect is similar, but rather less pronounced, for ATLAS dijet data.

**Fig. 31:**
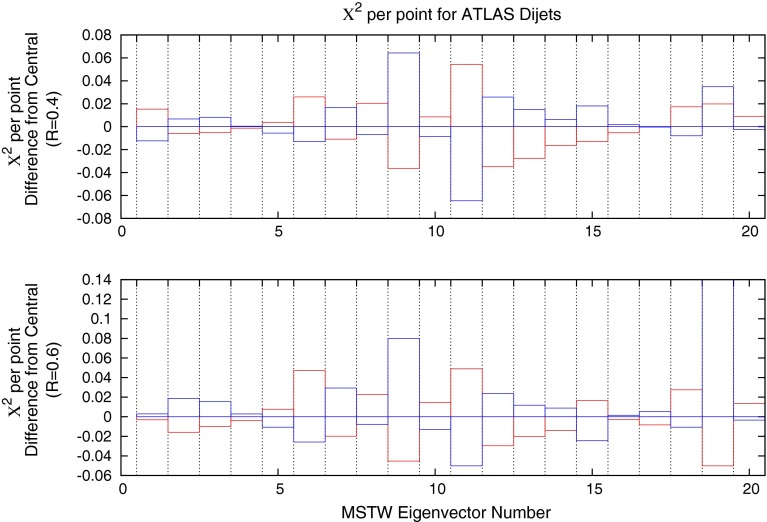
Deviations in fit quality from the MSTW 2008 NLO central value for each of the 20 eigenvector directions. *Blue* (*red*) *bars* indicate the positive (negative) direction of deviations in the eigenvector dimension

### Effect on MSTW PDFs

Figure 31 shows the change in the $\chi ^2$ for each eigenvector direction of the MSTW 2008 NLO set for the ATLAS dijet data, using 68 % confidence levels and a scale choice of $2p_T$. The plots show that, for the majority of the eigenvectors, a direction may be chosen in which the fit quality may improve, if only slightly. The eigenvector which contributes most significantly across the inclusive- and dijet datasets is number $9$, which is almost exclusively influenced by the gluon PDF. The other biggest contributors are influenced by a more mixed set of PDFs.

**Fig. 32:**
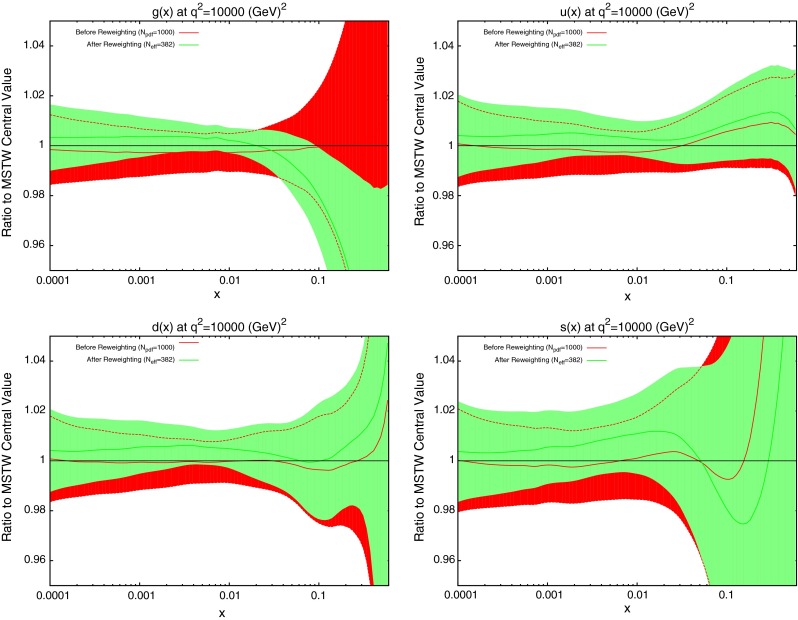
Effect of PDF reweighting on the gluon, up, down and strange distributions for DØ dijet data

Next the reweighting procedure used in the previous section is repeated for the dijet datasets. The results for DØ dijets are shown in Fig. 32. The scale choice used in the plots shown is $M_\mathrm{JJ}$, however, it was observed that a very similar effect was seen for the other two scale choices. Whilst the value of $N_\mathrm{eff}$ changes from 382 in the shown plots to 166 for $p_T^{av}$ and 56 for $M_\mathrm{JJ}/0.7\cos { h}(y^*)$, the actual reweighted PDFs move in the same directions. All of the parton densities here are affected to some degree. Notable is the fact that there is a reasonable shift from the central values, especially for the gluon which also sees an improvement in the error band at the previous noted $x$ region. Given that the DØ inclusive jet data is included in the MSTW fit, this could be motivation to also attempt an inclusion of dijet data. The general trend of a larger gluon at low $x$ and lower at high $x$, along with slightly larger quark densities overall is similar to that of the ATLAS and CMS inclusive jet data shown in the previous Section.

**Fig. 33:**
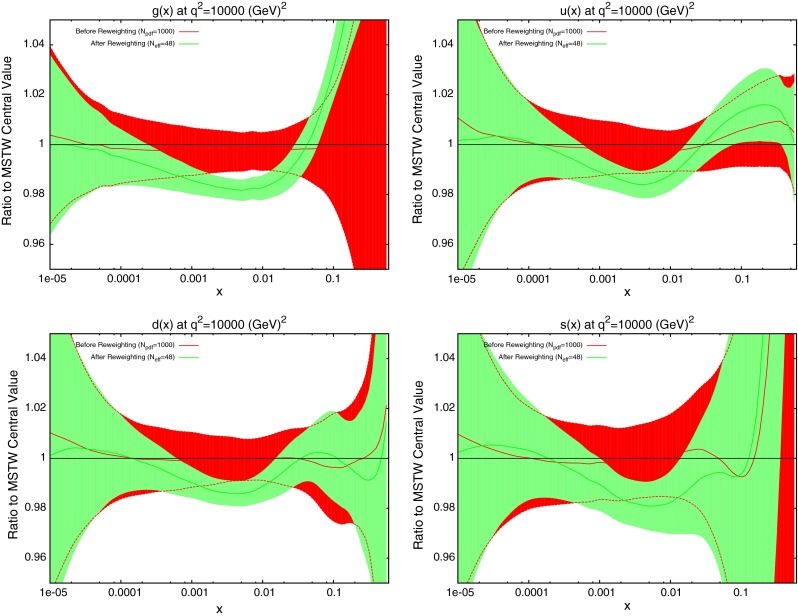
Effect of PDF reweighting on the gluon, up, down and strange distributions for ATLAS dijet data. The scale choice used is $M_\mathrm{JJ}$

**Fig. 34:**
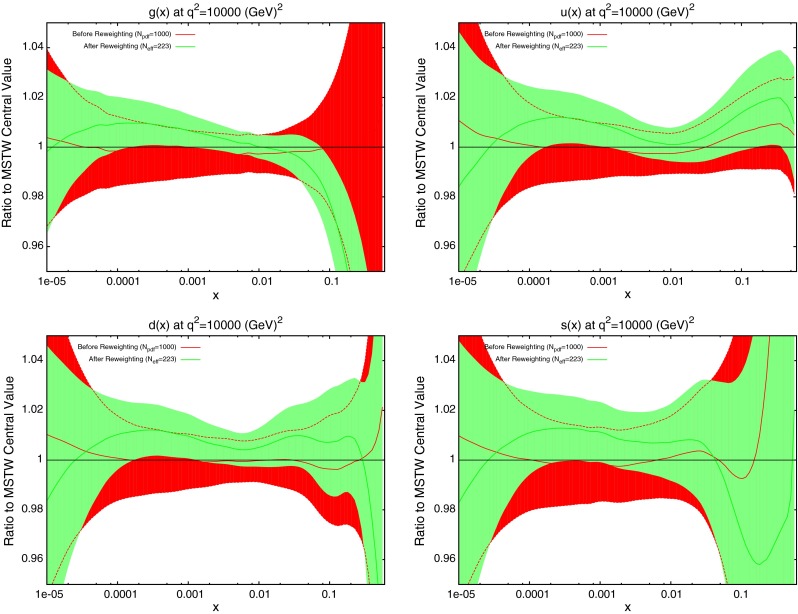
Effect of PDF reweighting on the gluon, up, down and strange distributions for ATLAS dijet data. The scale choice used is $2p_T^{av}$

Next, the PDFs are reweighted using the ATLAS dijet data. This time, a difference in the PDF effect is observed between the different scale choices, which indicates a fundamental difference in the implied physics. For the choice of $M_\mathrm{JJ}$, shown in Fig. 33, the gluon is moved well below its error band at moderate $x$ values, and above it at high $x$. All of the quark PDFs are also significantly shifted with a reduction in error band size. For the other two scale choices, shown in Fig. 34 for $2p_T^{av}$ and very similar for $M_\mathrm{JJ}/0.7\cos { h}(y^*)$, a less drastic and contradictory behaviour is seen, with the reweighted PDFs generally not moving outside of the error bands and the main effect being the softening of the gluon at high $x$. All of the reweighted PDFs give an improved fit to data from the standard MSTW predictions: $M_\mathrm{JJ}$ changes from 2.30 to 1.95 per point, whilst $2p_T^{av}$ moves from 1.98 to 1.90. However, the value of $N_{eff}$ is very low for the $M_\mathrm{JJ}$ and $M_\mathrm{JJ}/0.7\cos { h}(y^*)$ calculations, i.e. well under 100, and so the results should be considered with due care. Any value below 100 implies either that the original fit is very incompatible or the data is extremely constraining and hence that the reweighting is having a very large effect and is therefore not fully reliable. Without any clear preference for one of the particular scale choices it is difficult to conclude that the true effect on the PDFs from the ATLAS dijet data is within any PDF variation spanned by any of the scale choices. For the gluon this is then wider than the original uncertainty band. All choices seem to favour a slightly larger up quark distribution at high $x$, as does the DØ dijet data. Note that in each of these reweighting exercises the data normalisation moves by no more than one standard deviation.

### CMS dijet data

**Fig. 35:**
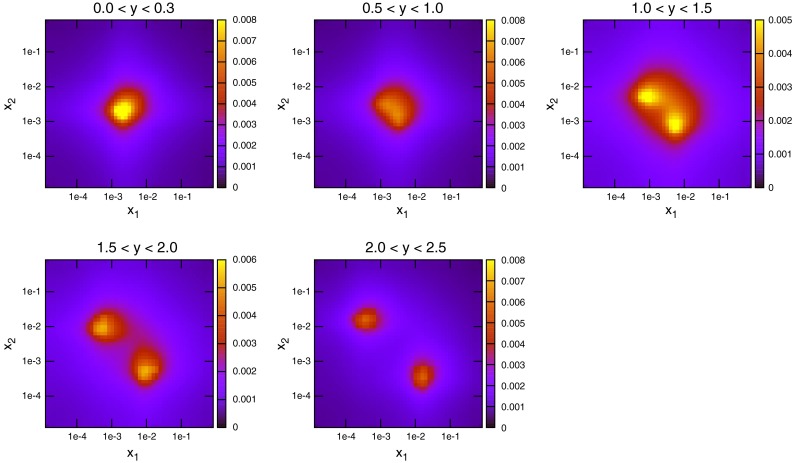
Values of $x_1$ and $x_2$ for each event generated in NLOJet++ for CMS dijets

Finally, as with the inclusive jets cross sections, the most recent and highest precision published dijet data has come from the CMS experiment [29]. The data consists of 54 points binned in $M_\mathrm{JJ}$ and $y_\mathrm{max}$. This is significant since it is the same rapidity binning as DØ, and different from ATLAS. Now any differences between the two approaches can be compared at the same collider. The $x$ distributions of NLOjet++ events generated for this dataset are shown for each rapidity bin in Fig. 35. Due to the rapidity definition being the same as that at the Tevatron, the distribution resembles Fig. 16, except with generally lower values of $x$ probed. Here, central dijets are probed at around $x\sim 0.005$ for lowest $M_\mathrm{JJ}$, with the highest-rapidity dijets reaching $x\sim 0.0001$. The data in this case extends less far in rapidity, from $y_\mathrm{max}=0$ to $y_\mathrm{max}=2.5$, than the ATLAS data, which went all the way to $y^*=4.4$. Although the definitions are different, it must be true that the ATLAS data includes higher-rapidity jets, since $y^*$ is defined as half the difference of the dijets’ rapidities, and so the highest bin necessarily only includes two very high-rapidity jets. Due to the low-rapidity cutoff, the issue of scale choice should be expected to not be as important, since all of the data is in the region where the $p_T^{av}$ choice behaved normally for ATLAS jets.

**Fig. 36:**
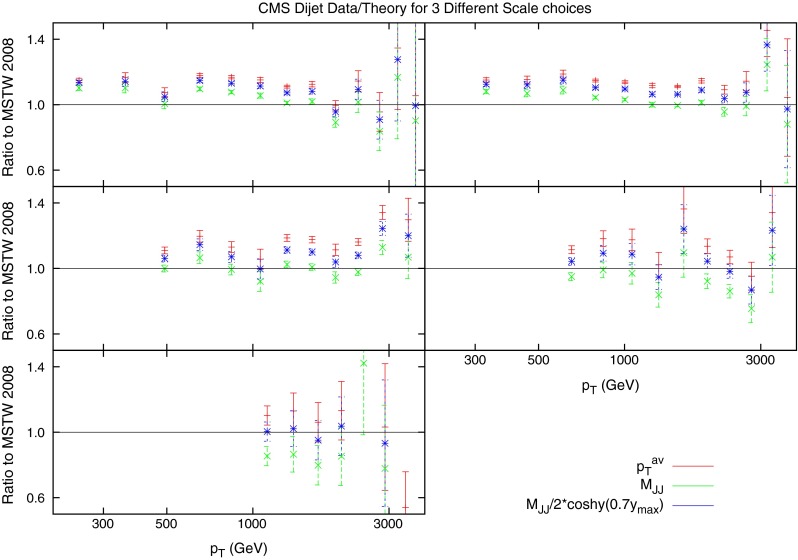
Ratio of data to theory for CMS dijets for all rapidity intervals. All three of the scale choices discussed are shown

**Fig. 37:**
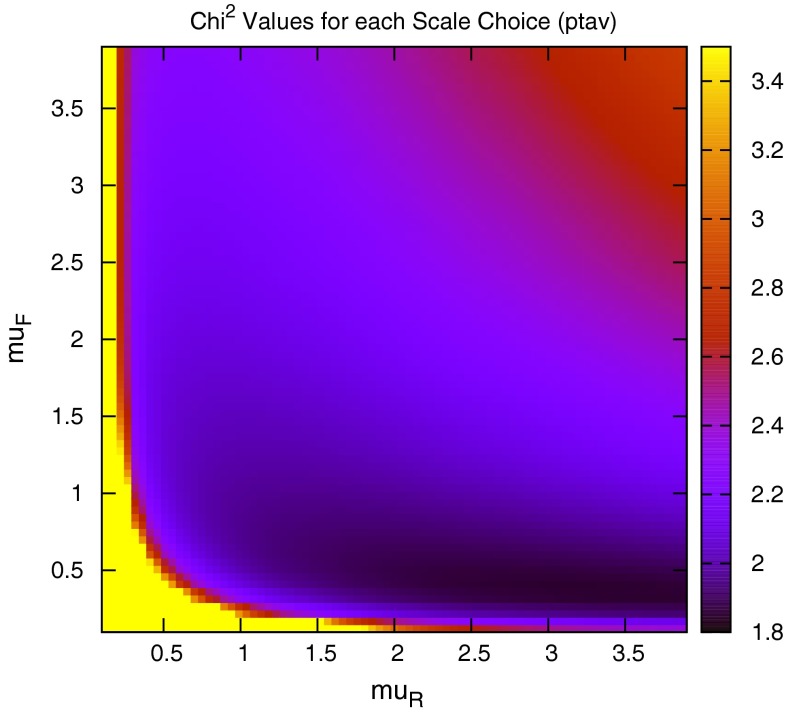
$\chi ^2$ value for every combination of $\mu _R$, $\mu _F$ for CMS dijets

The ratio of data to theory for the three scale choices is shown in Fig. 36. The scale variation has much less of an effect than for the ATLAS dijets, mostly due to the fact that the rapidity cut off is much lower, and the region where the most deviation occurred in the ATLAS dijets is avoided. The variation of the $\chi ^2$ fit with the scales for the $p_T^{av}$ calculation is shown in Fig. 37. Again, there is a region in the bottom left where the fit quality diverges exponentially, however, this region is much smaller than for the ATLAS dijets, again because of the lack of the high-rapidity region, where the calculation is known to behave peculiarly. The results are summarized in Table 13.

**Fig. 38:**
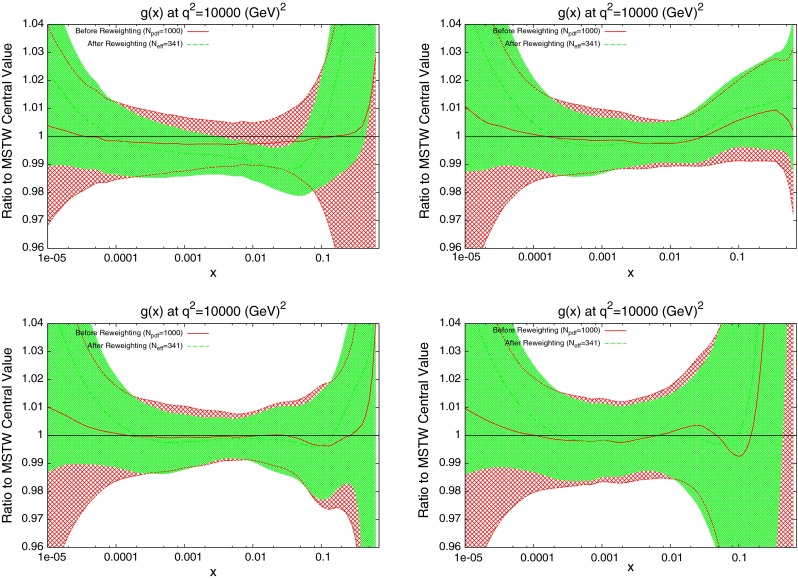
Effect of PDF reweighting on the gluon, up, down and strange distributions for CMS dijet data. The scale choice used is $p_T^{av}$

The results of the PDF reweighting are shown in Fig. 38. Only the plot for the scale choice $p^T_{av}$ are shown, since for this dataset the three choices are all in general agreement, unlike for the ATLAS dijets. The shape of the reweighted gluon is similar to that of the $M_\mathrm{JJ}$ ATLAS dijets, with a smaller gluon at moderate $x$ preferred, though the change is smaller than this, i.e. within the PDF uncertainty. This effect is in contradiction to the preferred gluon of the inclusive jet data, implying a conflict between the two datasets. However, it may be that higher-order corrections beyond NLO in QCD (or electroweak corrections) do not have exactly the same shape dependence, and this could potentially remove, or reduce any tension between the constraints from inclusive and dijet data.

## Direct inclusion of inclusive data in PDF fits

In this section, new PDF sets are produced including the LHC inclusive jet data directly into PDF fits using the MSTW2008 framework. There was sufficient motivation from the eigenvector reweighting studies into the ATLAS combined 2.76 and 7 TeV data and the CMS data to justify this. In addition, this is an opportunity to further test the validity of the reweighting technique as a general method of quantifying the effect of a new dataset on PDFs. Two fits are performed in this section, the first of which includes only the ATLAS 7 TeV and CMS inclusive data, both of which were calculated with FastNLO version 2. The second fit additionally includes the ATLAS combined data, which is calculated using APPLgrid and required further modifications to the fitting code.

### Fit with ATLAS 7 TeV and CMS inclusive jet data

In order to include the CMS data into an MSTW fit, the first necessary task was to modify the fit code to include FastNLO version 2 [19]. This new version allows more scale flexibility within the cross section calculation. The fit is performed allowing the same parameters to be free as in the standard MSTW2008 set. Initially, $\alpha _s(M_Z^2)$ was allowed to be free, and a reasonable improvement in the global fit from 2,795 to 2,781 over 2,922 data points was obtained. This fit, however, included a decrease in $\alpha _s(M_Z^2)$ from 0.1202 to 0.1189, which caused much of the improvement. Subsequently, in order to properly quantify the effect on just the PDFs, $\alpha _s(M_Z^2)$ was held fixed. This fit yielded a smaller improvement of only eight points to 2,786.

The effect on each dataset included in the fit is shown in Table 14. The ATLAS and CMS $\chi ^2$ values for MSTW2008 were first calculated using the fitting code by passing through the central value and bypassing the minimisation steps. Once they are included in the minimisation, a large improvement in the fit to CMS data is seen with a more modest improvement for the ATLAS data. The fact that both datasets prefer a smaller $\alpha _s(M_Z^2)$ is shown in the fact that the improvement is less pronounced when it is held fixed. In general, the fit to the various DIS datasets is left unchanged by both of the new fits. Interestingly, the Tevatron inclusive jet fits worsen very slightly with the inclusion of the LHC scenarios, although on the whole the Tevatron data remains also unchanged. The improvement of the global fit with $\alpha _s(M_Z^2)$ free can be understood through the stark improvement in the BCDMS proton $F_2$ measurement. This set returns to its original $\chi ^2$ value once $\alpha _s(M_Z^2)$ is fixed. These PDFs will be named here MSTWCMS, due to the dominance of the CMS inclusive jet data on the improvement in fit quality.

**Fig. 39:**
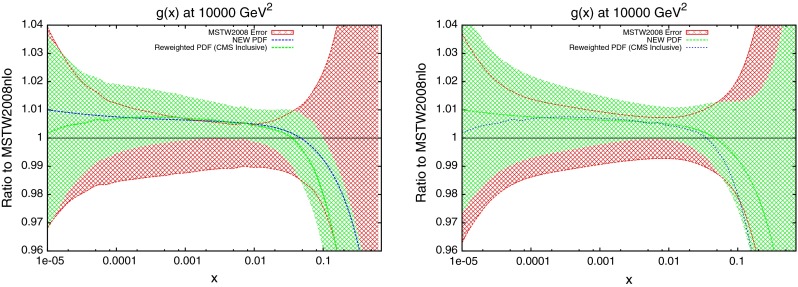
Comparison of the gluon for standard MSTW fit, reweighted PDF (using CMS inclusive jets to reweight), and the new fit directly including the ATLAS and CMS data. All three central values are shown on each plot; the first compares the *error bands* for MSTW against reweighting, and the second compares standard MSTW to the new fit

The new central PDF is shown in Fig. 39, along with the reweighted PDF using the CMS inclusive data. The two error bands shown are the original MSTW2008 68 % confidence level, and the reweighted standard deviation of the randomly generated PDFs. It is clear that the new PDF requires a similar behaviour in the gluon as the reweighting technique. Whilst the two central lines to not exactly match, there is a trend for a $\sim$1 % increase in the gluon for much of the $x$ range, which turns into a rapidly decreasing gluon at around $x\sim 0.1$. The error band of the reweighted PDF is in good agreement with that of the new fit for most values of $x$. The only region with disagreement is at high $x$, where the reweighting technique appears to underestimate the error. Upon inspection of the top weighted PDFs used, all require a steeply falling gluon compared to MSTW2008, and so the standard deviation shows a strong grouping around this trend.

**Fig. 40:**
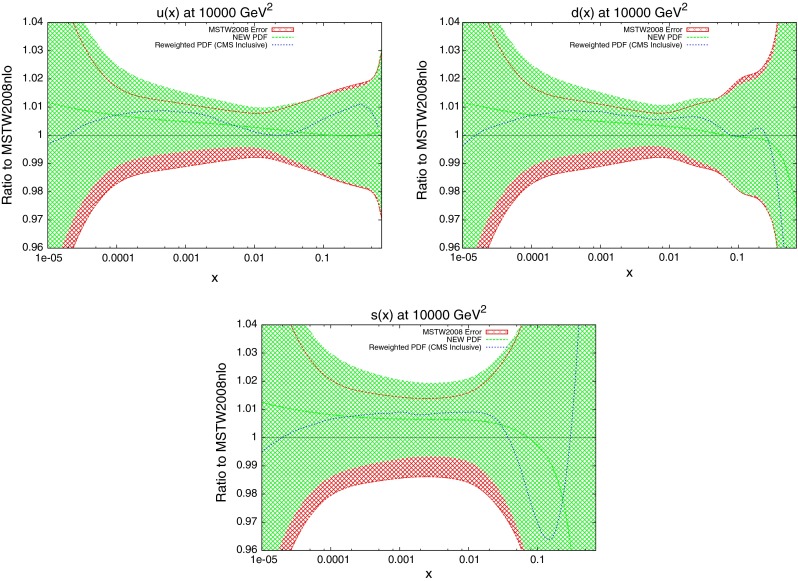
Ratio of the MSTWCMS quark distributions to MSTW2008. The central value of the reweighted PDF using CMS inclusive data is also shown for comparison

The new quark PDFs are shown in Fig. 40. These were shown to be important for the CMS inclusive jet data due to the $x$ values and resulting partons probed. The magnitude of change from MSTW2008 is comparable to the gluon, lending further evidence for the importance of these data. Again, there is good agreement between the reweighting technique and the direct inclusion of data. The only significant disagreement is in the high-$x$ strange distribution where the uncertainties are very large.

**Fig. 41:**
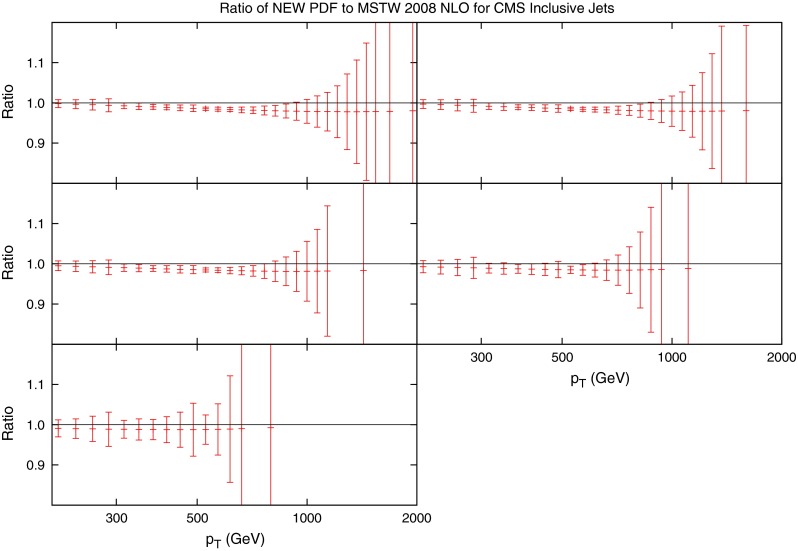
Ratio of CMS inclusive jet cross section predictions for the new PDFs and the standard MSTW 2008 PDFs

The new prediction for CMS inclusive jets is shown in Fig. 41. There is no change in shape between the new prediction and the MSTW2008 prediction, and most points lie within the experimental error bars. However, a systematic downward shift of $\sim$1 % is seen across most higher-$p_T$ data points. For lower-rapidity bins, where the experimental error is smallest, this shift brings some points out of agreement with the MSTW2008 prediction, and this is where the largest change in $\chi ^2$ originates.

### Eigenvectors

**Fig. 42:**
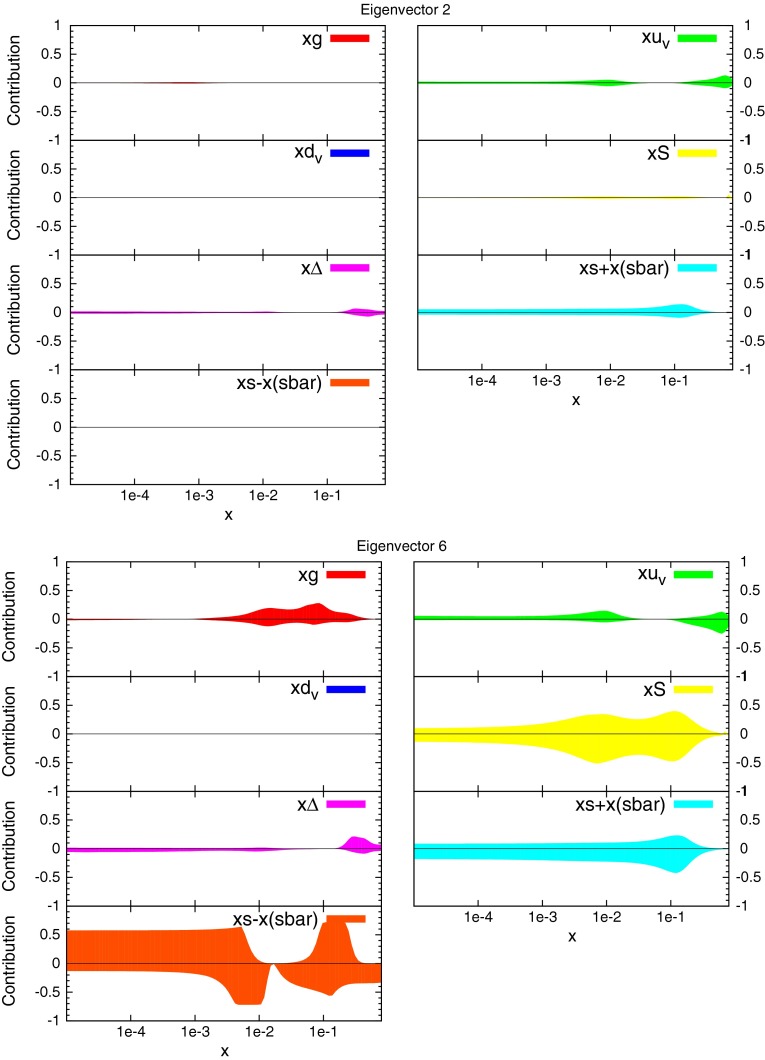
Fractional contribution to the uncertainty on major distributions from each eigenvector. Eigenvector 2 and 6 shown

**Fig. 43:**
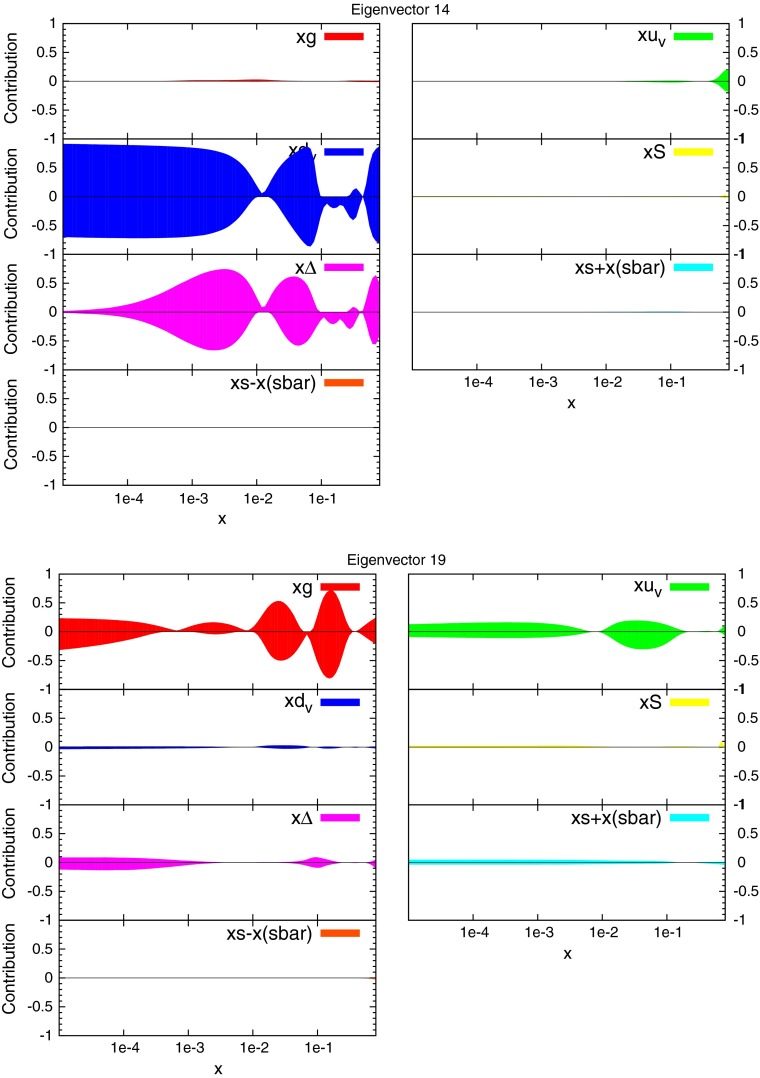
Fractional contribution to the uncertainty on major distributions from each eigenvector. Eigenvector 14 and 19 shown

The eigenvectors for the new fit are calculated in the same manner as the usual MSTW global fits. There are again 20 eigenvectors due to the same parameters being free, however, the dependence of each eigenvector on the underlying parameters and datasets has changed. The fractional contribution to the total uncertainty on selected distributions from some eigenvectors is shown in Figs. 42 and 43. These can be interpreted as the sensitivity to the underlying PDFs of each eigenvector, and can be compared to the equivalent plots for the MSTW2008 fit presented in [3].

**Table 13:** $\chi ^2$ values for CMS dijets

	$0.5*p_T^{av}$	$1.0*p_T^{av}$	$2.0*p_T^{av}$
MSTW2008 NLO	2.76	1.97	2.18

**Table 14:** Table of $\chi ^2$ values for each dataset included in the fits for the standard MSTW 2008 NLO fit and the new NLO fits with ATLAS 7 TeV and CMS data. The ATLAS and CMS values are quoted for MSTW 2008 despite not being included in the fit. These are simply the $\chi ^2$ values obtained when the fit code is run using the standard set without minimisation

Data set	MSTW2008	MSTWCMS $\alpha _s$ Free	MSTWCMS $\alpha _s$ fixed
BCDMS $\mu p$ $F_2$ [32]	182/163	172/163	182/163
BCDMS $\mu d$ $F_2$ [33]	190/151	188/151	189/151
NMC $\mu p$ $F_2$ [34]	121/123	122/123	120/123
NMC $\mu d$ $F_2$ [34]	102/123	103/123	102/123
NMC $\mu p/\mu d$ [35]	130/148	131/148	130/148
E665 $\mu p$ $F_2$ [36]	57/53	54/53	54/53
E665 $\mu d$ $F_2$ [36]	53/53	57/53	57/53
SLAC $\mu p$ $F_2$ [37, 38]	30/37	30/37	30/37
SLAC $\mu d$ $F_2$ [37, 38]	30/38	33/38	30/38
NMC/BCDMS/SLAC $F_L$ [32, 34, 39]	38/41	40/31	38/31
E866/NuSea pp DY [40, 41]	228/184	227/184	229/184
E866/NuSea pd/pp DY [42]	14/15	13/15	14/15
NuTeV $\nu N$ $F_2$ [43]	49/53	50/53	50/53
CHORUS $\nu N$ $F_2$ [44]	26/42	26/42	26/42
NuTev $\nu N$ $xF_3$ [43]	40/45	45/45	40/45
CHORUS $\nu N$ $xF_3$ [44]	31/33	32/33	31/33
CCFFR $\nu N\rightarrow \mu \mu X$ [45]	66/86	66/86	65/86
NuTeV $\nu N\rightarrow \mu \mu X$ [45]	39/40	39/40	40/40
H1 MB 99 $e^+p$ NC [46]	9/8	9/8	9/8
H1 MB 97 $e^+p$ NC [47]	42/64	43/64	44/64
H1 low $Q^2$ 96–97 $e^+p$ NC [47]	44/80	44/80	45/80
H1 high $Q^2$ 98–99 $e^-p$ NC [48]	122/126	122/126	120/126
H1 high $Q^2$ 99–00 $e^+p$ NC [49]	131/147	131/147	128/147
ZEUS SVX 95 $e^+p$ NC [50]	35/30	35/30	35/30
ZEUS 96–97 $e^+p$ NC [51]	86/144	86/144	85/144
ZEUS 98–99 $e^-p$ NC [52]	54/92	54/92	53/92
ZEUS 99–00 $e^+p$ NC [53]	63/90	63/90	62/90
H1 99–00 $e^+p$ CC [49]	29/28	29/28	29/28
ZEUS 99–00 $e^+p$ CC [54]	38/30	38/30	38/30
H1/ZEUS $ep$ $F_2^\mathrm{charm}$ [55]–[61]	107/83	106/83	109/83
H1 99–00 $e^+p$ incl. jets [62]	19/24	17/24	18/24
ZEUS 96–97 $e^+p$ incl. jets [63]	30/30	29/30	29/30
ZEUS 98–00 $e^\pm p$ incl. jets [64]	17/30	16/30	16/30
DØ II $p\bar{p}$ incl. jets [65]	114/110	116/110	115/110
CDF II $p\bar{p}$ incl. jets [66]	56/76	60/76	58/76
CDF II $W\rightarrow l\nu$ asym. [67]	29/22	30/22	29/22
DØ II $W\rightarrow l\nu$ asym. [68]	25/10	28/10	26/10
DØ II Z rap. [69]	19/28	17/28	19/28
CDF II Z rap. [70]	49/29	50/29	50/29
ATLAS 7 TeV incl. jets $(R = 0.4)$ [15]	(72/90)	66/90	70/90
CMS 7 TeV incl. jets [29]	(180/133)	163/133	169/133
Total	2,795/2,922	2,781/2,922	2,786/2,922

The CMS data itself directly constrains eigenvector 19 in this set. This can be seen to be almost entirely dependent on the gluon, although the up valence quark is also affected. Both distributions are most sensitive to this eigenvector in the higher-$x$ region, which is consistent with the conclusions of the reweighting study, where the gluon and quark distributions were shifted the most at high $x$ after reweighting to the CMS data.

**Fig. 44:**
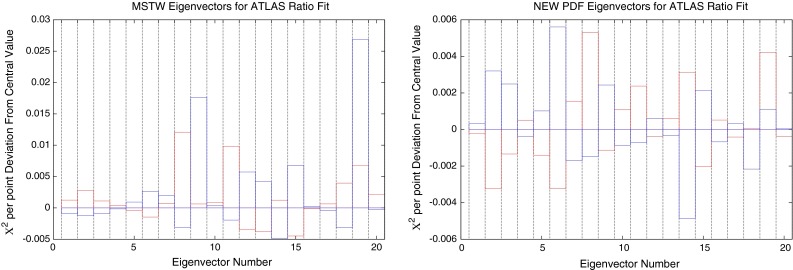
Change in fit quality to the ATLAS combined 2.76 and 7 TeV cross sections from the MSTW2008 (*left*) and MSTWCMS (*right*) central values for each eigenvector in the respective fits

The change in fit quality to the ATLAS inclusive jet combined data for each of the new eigenvectors is shown in Fig. 44 alongside the corresponding plot for MSTW2008. There is more dependence on the eigenvectors of the MSTW2008 set, and large increases in $\chi ^2$ can be obtained for many eigenvectors. The new eigenvectors do not produce this dramatic reduction in fit quality, implying a better agreement with the data. Despite this, there are still many eigenvectors which can improve the fit to a reasonable degree. The largest are eigenvectors 2, 6 and 14, shown in the figures. There are no longer any eigenvectors which give nearly such a large deterioration in fit quality, which shows that the CMS data has already provided much of the constraint possible from the combined ATLAS data in a completely compatible manner. Hence, the new PDF can then be said to provide a better fit to ATLAS combined data, with some scope still for further improvement.

### Reweighting of the new PDFs

**Fig. 45:**
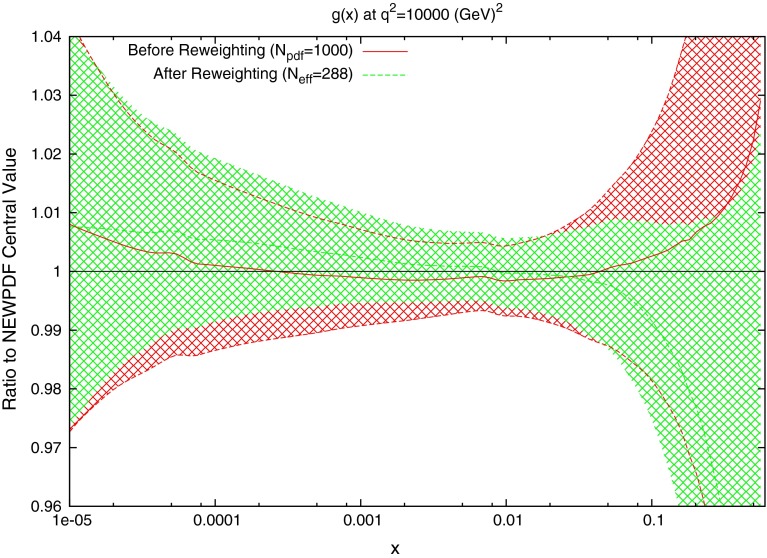
Reweighting of the new gluon PDF using ATLAS combined data

An important study which can now be performed is to reweight the new PDFs, which will again check the compatibility of the method with the standard fitting procedure. By using the new central value and eigenvectors, the $\chi ^2$ for ATLAS combined jets is calculated for 1,000 PDFs randomly generated in the eigenvector space. The distribution can then be compared to that of the PDFs randomly distributed in the standard MSTW eigenvector space. The observed effect is shown in Fig. 45. The cuts previously discussed are used along with the additive treatment of systematic errors. There is still a shift required of the gluon under reweighting. This can be interpreted as further evidence that the 7 TeV ATLAS inclusive data has little effect on the PDFs, and the combined data including the 2.76 TeV set must be used.

**Fig. 46:**
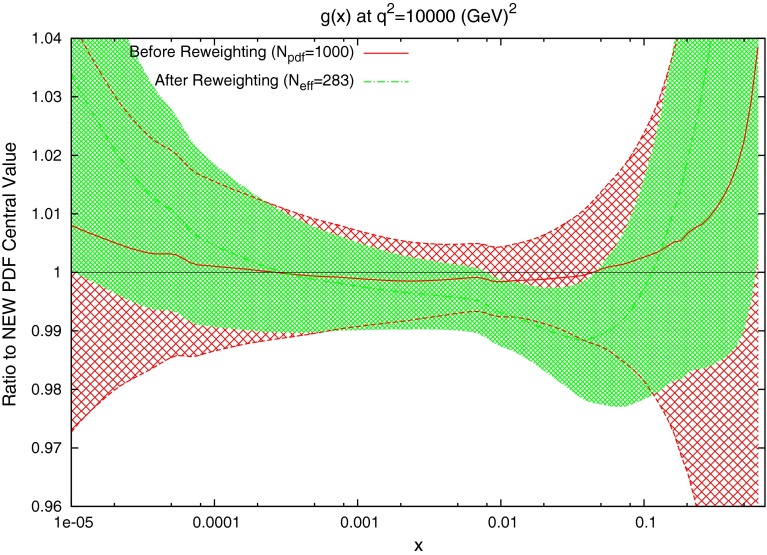
Reweighting of the new gluon PDF using CMS dijet data ($p_T^{av}$ scale choice)

Finally the dijet cross sections are studied using the new PDFs. After the previous studies which showed that in general the dijet datasets require a different shift in the PDFs to the equivalent inclusive jet data, this is the ideal test of compatibility between the data types. Figure 46 demonstrates the effect of the CMS dijet data on the new PDFs. There is in fact very little difference between the shape of the reweighted gluon with respect to the new PDF as that with respect to the MSTW2008 set. In fact, the slight reduction in the error band for the new PDF causes the reweighted central value to be marginally outside of the error band for a small $x$ range. The trend is still opposing the inclusive jet data, with a smaller gluon required at moderate $x$, and a larger gluon at low $x$. The reweighted PDF has a $\chi ^2$ of 1.77 per point, compared to the unweighted central value which is 2.02 per point. Both of these values are larger than the 1.67 per point which is the value after reweighting to the MSTW2008 PDFs, which implies that the new PDFs are in fact slightly worse at describing the CMS dijet data, despite the corresponding inclusive jet data being newly included in these sets.

### $\Delta \chi ^2=1$ treatment

Until now, the reweighting procedure used in this article and in the previous MSTW study have used the standard MSTW eigenvectors, which are defined using dynamical tolerance levels. In this procedure, some eigenvectors are allowed to move further from the global minimum in $\chi ^2$ than others, depending on the deterioration in the fit quality to individual datasets in the relevant direction. This practice is similar to that used in the CTEQ/CT PDF determination, and though the NNPDF determination of PDFs uses a very different approach to determine the uncertainties, where a particular $\Delta \chi ^2$ is difficult to identify, the PDF uncertainties from MSTW and NNPDF (and CT10), are very similar; see e.g [71, 72]. However, when reweighting using the eigenvectors, it may be interesting to consider the use of a set tolerance of $\Delta \chi ^2=1$ in each direction instead, i.e. the conventional “textbook” choice.

**Fig. 47:**
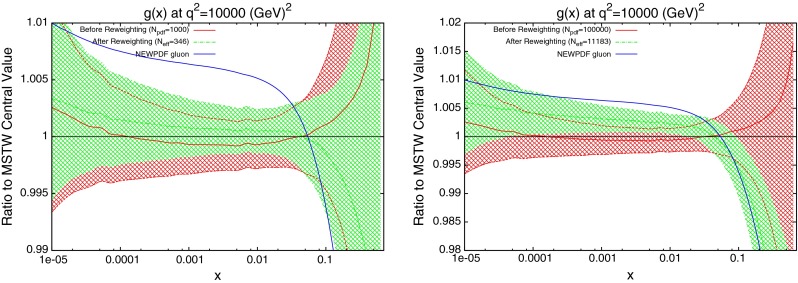
Reweighted gluon using CMS inclusive data, the $\Delta \chi ^2=1$ error treatment and 1,000 PDFs (*left*) or 100,000 PDFs (*right*). The reweighting formula is a pure exponential

The reweighted gluon using this technique is shown with the gluon of the new fit PDF in the left of Fig. 47. Here we also test the hypothesis that when using the “textbook” method for uncertainty determination the appropriate reweighting function is a pure exponential. In fact the results do not seem strongly dependent on the reweighting function used. Whilst the reweighting had previously agreed well with the required shift for the new PDF, there is clear disagreement here. The new PDF is well outside the $1 \sigma$ error band. This can be explained simply by an inability for the random PDFs to be generated in the required range. Given that, on average, the dynamic tolerance levels for the eigenvectors in the MSTW2008 fit are approximately 3–4, by rescaling to a value of 1, we can assume that all error bands and fluctuations will be reduced by a factor of 3 or 4.

**Fig. 48:**
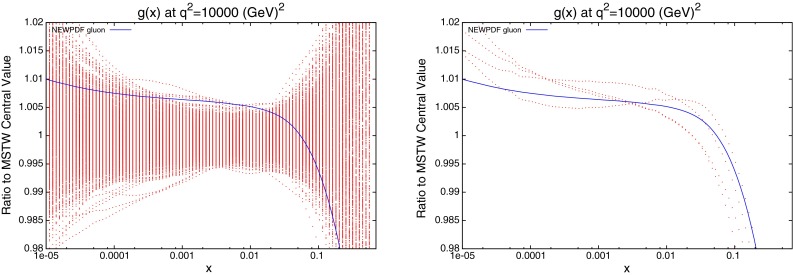
Plot of the 1,000 randomly distributed PDFs under the $\Delta \chi ^2=1$ prescription (*left*) and the highest weighted of 100,000 randomly distributed PDFs under the $\Delta \chi ^2=1$ prescription (*right*)

The 1,000 randomly distributed PDFs using the $\Delta \chi ^2=1$ method are shown in the left of Fig. 48, and demonstrate this inability to replicate the new PDF gluon. Whilst a very small handful extend to the required upward shift, these are drowned out by the vast majority which, whilst weighted lightly, contribute the most to the reweighted PDF. Indeed, very few of these 1,000 PDF sets give a gluon distribution which is similar to that required by the full global fit including the CMS jet data (let alone also a set of quark distributions of exactly the correct shape). This is not that surprising since the deterioration of the other data in the fit is a few units, and the new gluon is 2–3$\sigma$ from the MSTW2008 gluon if $\Delta \chi ^2=1$ is used as the uncertainty criterion. Hence, 1,000 random PDFs sets is very likely not enough to produce a significant number near the best fit, and hence to provide a correct reweighting procedure.

Hence, we repeat the exercise using 100,000 PDF sets.In the right of Fig. 48 we show the highest weighted of these PDF sets. Even with this number of random PDFs only a small number have a gluon of very nearly the ideal shape. In the right of Fig. 47 we show the reweighted gluon using 100,000 PDF sets. Clearly this is different from that with 1,000 sets and is much nearer to that obtained with the full fit. It does not appear as though even this number of sets has led to convergence, but it is impractical to generate an even larger number of PDFs. However, we can make two conclusions from this study. If the true PDF modification is well outside what is defined to be the uncertainty band of the PDF the reweighting procedure becomes very inefficient. We also conclude that when using the conventional MSTW uncertainty prescription the CMS inclusive jet data is compatible with the MSTW2008 PDFs at about the one $\sigma$ level, and hence has a significant, but not dramatic effect on new PDFs, whereas using the “textbook” uncertainty determination the CMS data is quite distinctly incompatible with the MSTW2008 set, and by inference with some of the data used in the PDF determination. Similar size changes in PDFs and in $\chi ^2$ have frequently been observed when adding new datasets to the PDF fit, but the reweighting procedure allows us to illustrate the results using this particular new set in a new manner.

### Direct Inclusion of ATLAS 2.76 + 7 TeV Data

The final new fit performed in this study is to include the ATLAS 2.76 TeV data in conjunction with the already present 7 TeV data. Whilst FastNLO tables for the 7 TeV data are available, this is not the case for the 2.76 TeV data. This presents the opportunity to interface APPLgrid, which did not exist at the time of the MSTW2008 fit, into the MSTW fitting code. Due to the fact that the MSTW code uses by default additive errors, the stringent cuts on the ATLAS ratio data discussed previously were applied to the dataset in the fit. APPLgrid grids were used for both of the ATLAS cross sections, and FastNLO was kept for all of the other jet cross sections, including the CMS inclusive jet data introduced in the previous section.

Again, $\alpha _s(M_Z^2)$ is allowed to initially go free, yielding an improvement of 20 fit points, and yielding a new value of $\alpha _s(M_Z^2)=0.1187$. Most notable in this fit, shown as the second column in Table 15, is the very significant improvement in the ATLAS combined jet data. This improvement mostly goes away after holding $\alpha _s(M_Z^2)$ fixed at its MSTW2008 value. The total improvement in $\chi ^2$ in this case from the MSTW2008 NLO fit is 13 points, and so is better than the previous fit which only included the CMS and ATLAS 7 TeV data. The majority of the improvement is again caused by the CMS data which reduces by 14 points. The ATLAS combined data improves by 4 points, better than the 2 by the ATLAS 7 TeV data in the previous fit, however, there are 20 more points in the combined dataset. This fit will be named here as MSTWATLAScomb, due to the additional inclusion of the combined ATLAS data. The fact that the CMS data improves more in this fit than the last demonstrates the excellent compatibility between the two LHC datasets. The ATLAS combined data, whilst only improving a small amount, is clearly having an additional affect on the global fit in the same direction preferred by the CMS data. The increase in global $\chi ^2$ and the most constraining eigenvector is shown in Table 16. This is similar to the fit adding the CMS data alone, though the nominal order of the eigenvectors is altered slightly due to small changes in the size of the eigenvalues with the additional ATLAS jet data.

**Fig. 49:**
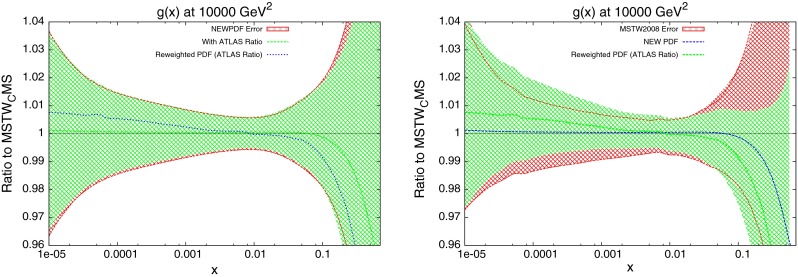
Comparison of the gluon for the CMS fit, reweighted PDF (using ATLAS Ratio jets to reweight), and the new fit directly including the ATLAS Ratio and CMS data. The central values are the same on both plots; however, the first plot shows the new PDF’s *error band* *in* *green*, whilst the second shows the reweighed PDF’s *error band in green*

Finally, the reweighting procedure can be once again tested against the direct inclusion of a new dataset. This is achieved by reweighting the new MSTWCMS PDFs using the ATLAS combined data. When comparing this to the change in the gluon by moving from MSTWCMS to MSTWATLAScomb, the results should agree if the two methods are consistent. The results for the gluon are shown in Fig. 49. The agreement between the two methods is not as obvious as in the previous case with the inclusion of the CMS data, however, the general trends are comparable, and both agree within their respective error bands. The MSTWATLAScomb fit is almost identical to MSTWCMS for most of the $x$ range, with the only divergence coming at high $x$ where the uncertainties are highest. This is testament to the dominance of the CMS data in both fits. The ATLAS combined data has limited effect on its own when additionally added to the CMS fit. The left plot in Fig. 49 shows that there is a small improvement in the error band of the PDFs when including the ATLAS ratio data, and so there is a benefit to including both datasets simultaneously.

## NNLO PDFs

When considering NNLO PDFs, it is strictly necessary to use NNLO matrix elements for the theoretical predictions. For hadron–hadron inclusive jet cross sections these calculations have to date not been produced, and so approximations must be utilised to obtain the theoretical cross sections. The approximation used in the MSTW2008 analysis for Tevatron inclusive jets is based on the calculation by Kidonakis and Owens [73]. This calculation produces a threshold resummation which is based on the assumption that the parton–parton scattering phase space is restricted to the threshold region of $x_T=2p_T/\sqrt{s}\sim 1$, due to the rapid decrease in PDFs at high $x$. The corrections are provided within the FastNLO framework, and so have been included for the use of Tevatron inclusive jet data in NNLO fits. These corrections have recently been reproduced in [74]. In the latter article a comparison is also made between the threshold approximation and the full NLO result, observing that best agreement is for low values of cone radius $R \sim 0.3$–$0.4$. This suggests that the threshold approximation of the NNLO corrections may be a little low for the $R$ values more like $R=0.7$ used at the Tevatron, though this is not definite, and as discussed more below, these corrections are quite small. A more detailed threshold calculation has also recently been performed in [75], where good agreement is seen between the threshold approximation and the full NLO calculation at high $p_T$ independent of $R$. However, despite a large $R$ dependence at NLO, the further correction from NLO to NNLO shows much less dependence, and is often of order $15~\%$ if the scale choice is $p_T$. Unfortunately these results are not yet in a form which can easily be incorporated in a PDF fit.

**Table 15:** Table of $\chi ^2$ values for each dataset included in the fits for the standard MSTW 2008 NLO fit and the new NLO fits with CMS and ATLAS combined 2.76 and 7 TeV data. The ATLAS and CMS values are quoted for MSTW2008 despite not being included in the fit. These are simply the $\chi ^2$ values obtained when the fit code is run using the standard set without minimisation

Data set	MSTW2008	MSTWATLAScomb $\alpha _s$ Free	MSTWATLAScomb $\alpha _s$ fixed
BCDMS $\mu p$ $F_2$	182/163	170/163	182/163
BCDMS $\mu d$ $F_2$	190/151	189/151	190/151
NMC $\mu p$ $F_2$	121/123	123/123	119/123
NMC $\mu d$ $F_2$	102/123	103/123	101/123
NMC $\mu p/\mu d$	130/148	131/148	129/148
E665 $\mu p$ $F_2$	57/53	53/53	54/53
E665 $\mu d$ $F_2$	53/53	57/53	57/53
SLAC $\mu p$ $F_2$	30/37	30/37	30/37
SLAC $\mu d$ $F_2$	30/38	33/38	29/38
NMC/BCDMS/SLAC $F_L$	38/41	40/31	38/31
E866/NuSea pp DY	228/184	227/184	228/184
E866/NuSea pd/pp DY	14/15	13/15	14/15
NuTeV $\nu N$ $F_2$	49/53	50/53	50/53
CHORUS $\nu N$ $F_2$	26/42	26/42	26/42
NuTev $\nu N$ $xF_3$	40/45	45/45	40/45
CHORUS $\nu N$ $xF_3$	31/33	32/33	31/33
CCFFR $\nu N\rightarrow \mu \mu X$	66/86	67/86	65/86
NuTeV $\nu N\rightarrow \mu \mu X$	39/40	49/40	40/40
H1 MB 99 $e^+p$ NC	9/8	9/8	9/8
H1 MB 97 $e^+p$ NC	42/64	42/64	44/64
H1 low $Q^2$ 96–97 $e^+p$ NC	44/80	44/80	45/80
H1 high $Q^2$ 98–99 $e^-p$ NC	122/126	122/126	119/126
H1 high $Q^2$ 99–00 $e^+p$ NC	131/147	132/147	127/147
ZEUS SVX 95 $e^+p$ NC	35/30	35/30	35/30
ZEUS 96–97 $e^+p$ NC	86/144	86/144	85/144
ZEUS 98–99 $e^-p$ NC	54/92	54/92	54/92
ZEUS 99–00 $e^+p$ NC	63/90	63/90	62/90
H1 99–00 $e^+p$ CC	29/28	29/38	29/28
ZEUS 99–00 $e^+p$ CC	38/30	38/30	38/30
H1/ZEUS $ep$ $F_2^\mathrm{charm}$	107/83	105/83	109/83
H1 99–00 $e^+p$ incl. jets	19/24	16/24	19/24
ZEUS 96–97 $e^+p$ incl. jets	30/30	29/30	29/30
ZEUS 98–00 $e^\pm p$ incl. jets	17/30	16/30	17/30
DØ II $p\bar{p}$ incl. jets	114/110	116/110	116/110
CDF II $p\bar{p}$ incl. jets	56/76	63/76	58/76
CDF II $W\rightarrow l\nu$ asym.	29/22	29/22	29/22
DØ II $W\rightarrow l\nu$ asym.	25/10	28/10	25/10
DØ II Z rap.	19/28	18/28	19/28
CDF II Z rap.	49/29	49/29	50/29
ATLAS 2.76 TeV and 7 TeV incl. jets ($R=0.4$)	(159/114)	144/114	155/114
CMS 7 TeV incl. jets	(180/133)	161/133	166/133
Total	2,882/2,946	2,862/2,946	2,869/2,946

**Table 16:** Table of $\Delta \chi ^2$ values for $68~\%$ confidence level uncertainty for each eigenvector and the most constraining datasets for the new NLO fits with CMS and ATLAS combined 2.76 and 7 TeV data

Eigenvector number	$+$ Direction $\sqrt{\Delta \chi ^2}$	Most constraining dataset	$-$ Direction $\sqrt{\Delta \chi ^2}$	Most constraining dataset
1	4.30	Zeus ep 95–00 $\sigma _r^{NC}$	3.40	H1 ep 97–00 $\sigma _r^{NC}$
2	3.90	NuTeV $\nu N \rightarrow \mu \mu X$	3.50	NMC $\mu d \,\,F_2$
3	2.20	CCFR $\nu N \rightarrow \mu \mu X$	1.30	NuTeV $\nu N \rightarrow \mu \mu X$
4	3.50	NMC $\mu n/p \,\,F_2$	2.30	E866/NuSea $pd/pp$ DY
5	2.20	NuTeV $\mu N \,\, xF_3$	1.55	NuTeV $\nu N \rightarrow \mu \mu X$
6	4.35	H1 ep 97–00 $\sigma _r^{NC}$	3.00	NuTeV $\nu N \rightarrow \mu \mu X$
7	2.05	DØ II $W \rightarrow l \nu$ asym.	2.80	BCDMS $\mu d F_2$
8	4.90	NuTeV $\mu N \,\,F_2$	1.90	BCDMS $\mu p \,\,F_2$
9	5.00	Zeus ep 95–00 $\sigma _r^{NC}$	3.90	H1 ep 97-00 $\sigma _r^{NC}$
10	2.95	DØ II $W \rightarrow l \nu$ asym.	3.25	SLAC $\mu p \,\,F_2$
11	4.80	CDF $p\bar{p} \rightarrow$ jets	4.05	H1 ep 97–00 $\sigma _r^{NC}$
12	5.45	NuTeV $\nu N \rightarrow \mu \mu X$	3.10	E866/NuSea $pd/pp$ DY
13	1.40	NuTeV $\nu N \rightarrow \mu \mu X$	3.35	E866/NuSea $pp$ DY
14	3.60	NMC $\mu d \,\,F_2$	3.50	NMC $\mu n/p \,\,F_2$
15	2.40	H1 ep 97–00 $\sigma _r^{NC}$	3.80	NuTeV $\mu N \,\,F_2$
16	2.05	CCFR $\nu N \rightarrow \mu \mu X$	1.10	E866/NuSea $pd/pp$ DY
17	1.60	E866/NuSea $pd/pp$ DY	2.70	NuTeV $\nu N \rightarrow \mu \mu X$
18	2.15	DØ II $W \rightarrow l \nu$ asym.	1.80	E866/NuSea $pd/pp$ DY
19	2.80	H1 ep 97–00 $\sigma _r^{NC}$	4.30	CMS $pp \rightarrow$ jets
20	5.30	NuTeV $\nu N \rightarrow \mu \mu X$	1.95	NuTeV $\nu N \rightarrow \mu \mu X$

As a check on reliability of NNLO results we have rerun the NNLO MSTW08 fit with the threshold corrections multiplied by quite an extreme factor of two. This results in a lowering of $\alpha _s(M_Z^2)$ by about 0.001 and a slightly higher gluon PDF at low $x$ and slightly smaller gluon at high $x$, with changes about one sigma or less. Hence, the change is not dramatic, and actually rather similar to the changes seen at NLO in this article which are induced by the LHC jet data. The fit quality does deteriorate, particularly for DØ data, but more due to details of shape rather than normalisation, i.e. the threshold corrections are unlikely to be exactly the correct shape in $p_T$, particularly at low-$p_T$ values, but the gluon distribution probed here is already very well constrained by the HERA DIS data. Similarly, removing the threshold factor entirely and performing an NNLO fit (the default procedure used by some groups in NNLO fits) results in a raising of $\alpha _s(M_Z^2)$ by about 0.001 and a slightly lower gluon PDF at low $x$ and slightly larger gluon at high $x$, with changes about one sigma or less. Simply using a constant $K$-factor of $15~\%$ changes the fit quality by only about one unit, and both PDFs and $\alpha _S$ change by much less than one standard deviation.

**Fig. 50:**
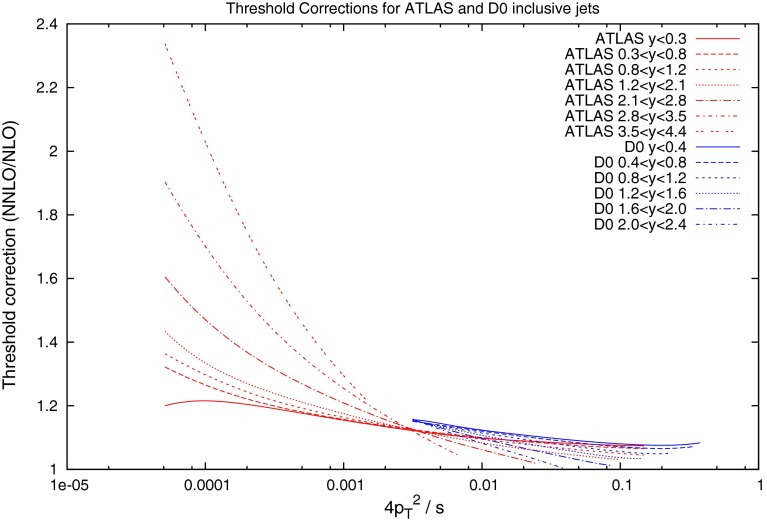
Comparison of NNLO threshold corrections for ATLAS and DØ inclusive jets as a function of $x_T^2=4p_T^2/s$

In order to help facilitate the inclusion of the LHC jet data into an NNLO fit the threshold corrections have also now been calculated for the new data and implemented in FastNLO for ATLAS data. The results are shown in Fig. 50, where the ATLAS data is presented alongside that of DØ (similar plots appear in [74], but none extending to such low-$p_T$ values or showing a range in rapidity values, and in [75]). The main point of note is that the LHC phase space spans a region which extends much further from the threshold region than the Tevatron. The Tevatron threshold corrections maintain a small correction of approximately $\sigma _\mathrm{NNLO}\sim 1.1$–$1.2 \sigma _\mathrm{NLO}$ across the majority of the phase space. However, this correction clearly increases away from threshold. The corresponding ATLAS calculation demonstrates that this trend continues even further, and although the central jets maintain a reasonable correction throughout, the forward jet corrections become very large with decreasing $x_T$.

It is clear that for LHC jets it will be necessary to include the full NNLO matrix elements in order to perform a full NNLO fit. Although not yet fully performed, the gluon–gluon process has been calculated by Gehrmann de Ridder et al. [76, 77]. These calculations have shown a NNLO results of between 1.1 and 1.3 times the NLO prediction across all jet $p_T$ values, and suggest that the threshold corrections indeed are not applicable to all LHC scenarios, i.e. at $p_T$ values such that one is very far from threshold. For inclusive jet cross sections at the Tevatron the threshold corrections seem reasonable when compared to the full NNLO corrections so far known, certainly when one considers the very large systematic uncertainties, including luminosity, which allow the data to move relative to theory.

In order to give some indication of how NNLO PDFs perform for LHC jet cross sections the NNLO PDF predictions for the ATLAS jet cross sections are calculated using just the NLO QCD cross section. The fit value with NNLO PDFs and NNLO coupling is shown in Table 17. The NNLO MSTW set describes the data after the low-$p_T$ cuts are applied well. Again the additive error treatment with the additional cuts is shown in the third column.We also repeat this exercise for the CMS inclusive jet data, and the results are in Table 18. Again the fit quality is very good. We have also tried, as a rough experiment, to compare the prediction using NNLO PDFs and a very approximate NNLO K-factor based on the results in [76, 77]. This causes the fit quality to deteriorate quite significantly if not dramatically.

**Table 17:** $\chi ^2$ per point for ATLAS combined data, both with and without $p_T$ cuts. The third column uses additive errors and has two additional anomalous points cut. The NNLO PDF set is used

	No cuts	HERAPDF cuts	Additive errors
MSTW 2008	1.32	0.927	1.44

**Table 18:** $\chi ^2$ per point (133 points) for NNLO PDFs for CMS inclusive jets

Scale	$p_T$
MSTW 2008	1.37

A refit of the PDFs results in a fit quality similar to that obtained in NLO fits (though slightly worse), little change in NNLO PDFs (though with a trend similar to the changes the LHC jet data induce in NLO PDFs) and a reduction in the NNLO $\alpha _S(M_Z^2)$ value extracted of order 0.001. However, since the gluon–gluon initiated contribution is not dominant, particularly at high $p_T$, it is difficult to draw strong conclusions beyond the fact that the still large systematic uncertainties on jet data at the LHC will likely allow fairly good quality fits for something similar to the current PDF and $\alpha _S$ values unless the full NNLO corrections are somewhat larger than seems likely.

## Conclusions

The data which has been measured during the first run of the LHC at 7 TeV is our first look at QCD in a new energy regime, and so the jet data is an important test of our current knowledge of PDFs. The conclusion from these first datasets is that the MSTW2008 PDFs hold up well in this regime, since none of the inclusive jet data from either ATLAS or CMS has required a PDF to move outside its 1$\sigma$ error band. The earliest released measurement was the least discerning for PDFs; the ATLAS inclusive jet cross section at 7 TeV using 36 pb$^{-1}$ of luminosity was inevitably dominated by systematics uncertainties. The fit quality obtained using MSTW2008 PDFs is very good and any variation in physics parameters used is incapable of improving the fit in any significant way. The lack of constraint due to large systematic uncertainties is significantly improved by the inclusion of a simultaneous measurement at centre of mass energy 2.76 TeV. The cancellation of systematic effects associated with jet energy scale provides a more suitable environment for testing PDFs. In this measurement, too, a good fit is found for MSTW2008 PDFs. The potential impact of the data was investigated using the PDF reweighting procedure. Although the data prefers a larger low-$x$ and softer high-$x$ gluon, these movements are still entirely within the error bands. A significant improvement in error is seen for the gluon across all $x$, which implies that, if included in a new fit, this data could provide more accurate PDFs for the LHC era. The published CMS inclusive data at 7 TeV is also analysed. With much higher luminosity than the ATLAS data, this is currently the published measurement with the most potential for PDF effects. Again a reasonable fit is found for MSTW2008, although the $\chi ^2$ per point is higher than the ATLAS fit. Due to the kinematics of the measurement, more focus is given to the quark densities for this set, and some reduction in the error bands is seen for all flavours. Again, including this data into a new fit would appear to provide PDFs with some improved constraints.

A detailed study into hadron–hadron dijet cross sections in relation to PDFs has also been presented. The instability of the calculation observed at the Tevatron using the scale choice of $p_T^{av}$ is explained by the behaviour of the kinematics at high rapidities. Calculations using other scale choices involving the dijet mass do not exhibit these problems, and so potentially provide a more reliable estimate of the scale uncertainty. For ATLAS dijets, the instability is even more clear for the $p_T^{av}$ calculation, with a very poor fit for low values of the scale multiplier quickly becoming an excellent fit for higher, unrealistic values. A study of the behaviour of the individual data points under scale variations demonstrates a saddle point structure which is centred around the central scale choice for low-rapidity bins, and which can become a constantly decreasing plane at higher rapidities. The best scale choice to maintain the stability of each bin under scale variations is something similar to $M_\mathrm{JJ}/0.7\cos { h}(y^*)$, as suggested many years ago [30]. The best fit to data is clearly obtained for scale choices similar to $\mu =M_\mathrm{JJ}$ for Tevatron data, whereas choices with less rapidity dependence are preferred by the ATLAS data. The difference is perhaps related to the fact that one is a proton–antiproton collider and the other a proton–proton collider, so different PDF combinations are probed even after one takes account of the different collider energies. The reweighting procedure has been conducted for DØ, ATLAS and CMS dijet data, and in general the resulting preferred PDF depends upon the scale choice used. This is not an ideal situation, since the physics cannot depend on an unphysical mathematical property of the calculation. However, for the CMS dijet cross section, which does not extend to very large rapidity, an agreement is reached between the scale choices, which is for a slightly smaller gluon across much of the $x$ range, except very high $x$, with the largest change at moderate $x \sim 0.05$ values. This also agrees with one of the scale choices for ATLAS dijets. This result is notable due to it being the opposite effect required to describe the ATLAS and CMS inclusive jet data, implying a possible conflict between the two datasets, or different forms of higher-order QCD corrections (the shape of the NNLO inclusive and dijet corrections so far available [76, 77] does not appear to be identical).

For the first time, LHC jet data has also been included directly in the framework of the MSTW PDF fit. The datasets included represent the highest-precision inclusive jet cross sections from both ATLAS and CMS to date. Two fits were initially performed with the new data, including the CMS inclusive jet data and ATLAS 7 TeV inclusive jet data: one allowing all standard MSTW parameters to be free, and one with $\alpha _s(M_Z^2)$ fixed to the MSTW2008 value. The entirely free set showed a significant reduction in global $\chi ^2$, although much of this was due to a shift in $\alpha _s(M_Z^2)$, which significantly improved some fixed target data. With $\alpha _s(M_Z^2)$ fixed the fit again improved, although to a lesser extent, with the majority of improvement coming from the fit to the new datasets. The improvement was dominated by the CMS data due to the previously noted issue of the large ATLAS systematic errors. With the new central values and eigenvectors, the reweighting procedure was applied to study the change in the effect after the addition of the ATLAS 2.76 TeV dataset. This was shown to still have an effect on the gluon, with a similar but less pronounced shape than was seen when reweighting the MSTW2008 set with the same data. Dijet data was shown again to have a different effect on the PDFs to the corresponding inclusive data, which is further evidence for their value in a future global fit. The ATLAS combined data, having shown an effect through reweighting, was then included in a second fit along with the CMS data. This further improved the global fit, with the fit to CMS showing a similar improvement to the first set, and an additional improvement from the ATLAS data itself. This demonstrated an excellent agreement between the ATLAS and CMS data sets, which had already been observed through the reweighting technique.
